# Multicenter Clinical Validation of the Molecular BD Max Enteric Viral Panel for Detection of Enteric Pathogens

**DOI:** 10.1128/JCM.00306-19

**Published:** 2019-08-26

**Authors:** William Stokes, Patricia J. Simner, Joel Mortensen, Margret Oethinger, Kathleen Stellrecht, Elizabeth Lockamy, Tricia Lay, Peggy Bouchy, Dylan R. Pillai

**Affiliations:** a Department of Medicine, University of Calgary, Calgary, Alberta, Canada; b Department of Pathology, Johns Hopkins School of Medicine, Baltimore, Maryland, USA; c Department of Pathology and Laboratory Medicine, Cincinnati Children’s Hospital, Cincinnati, Ohio, USA; d Providence Regional Laboratory, Oregon Region, Portland, Oregon, USA; e Department of Pathology and Laboratory Medicine, Albany Medical Center, Albany, New York, USA; f Center for Immunology and Microbial Diseases, Albany Medical College, Albany, New York, USA; g Becton, Dickinson and Company, BD Life Sciences, Sparks, Maryland, USA; h Becton, Dickinson and Company, BD Life Sciences, Quebec, Quebec, Canada; i Department of Pathology and Laboratory Medicine, Cumming School of Medicine, University of Calgary, Calgary, Alberta, Canada; j Department of Microbiology, Immunology, and Infectious Diseases, Cumming School of Medicine, University of Calgary, Calgary, Alberta, Canada; Memorial Sloan Kettering Cancer Center

**Keywords:** BD Max, enteric viral panel, gastrointestinal panel, adenovirus, astrovirus, enteric pathogens, norovirus, rotavirus, sapovirus

## Abstract

The conventional methodology for gastrointestinal pathogen detection remains time-consuming, expensive, and of limited sensitivity. The objective of this study was to evaluate the performance of the BD Max enteric viral panel (Max EVP) assay for identification of viral pathogens in stool specimens from individuals with symptoms of acute gastroenteritis, enteritis, or colitis. Prospective and archival stool specimens from adult and pediatric patients with diarrhea were collected in Cary-Blair medium or unpreserved containers. The results for specimens tested by the Max EVP (on the BD Max platform) were compared to those obtained by the reference method (alternate PCR assays, followed by bidirectional sequencing). Positive percent agreement (PPA) and negative percent agreement (NPA) were calculated. A total of 2,239 specimens were collected, with 2,148 being included for analysis. In this population, 39.6% of specimens were from outpatients, 42.1% were from patients <21 years old, and 49.7% were from females. Prevalence rates for prospective specimens were 7.3%, 4.5%, 3.5%, 2.4%, and 1.2% for norovirus, sapovirus, astrovirus, rotavirus, and adenovirus, respectively. PPA was 92.8%, 84.9%, 93.0%, 100%, and 95.6%, for norovirus, sapovirus, astrovirus, rotavirus, and adenovirus, respectively. NPA was ≥99.4% for all targets. In conjunction with the clinical presentation, laboratory findings, and epidemiological information, the Max EVP assay is effective for the differential diagnosis of enteric disease caused by norovirus, sapovirus, astrovirus, rotavirus, and adenovirus. This assay can be used individually for patients at high risk for a viral enteropathogen (e.g., in outbreak settings) or as an adjunct to other enteric bacterial panels.

## Abstract (precis)

The conventional methodology for gastrointestinal pathogen detection remains time-consuming, expensive, and of limited sensitivity. The objective of this study was to evaluate the performance of the BD Max enteric viral panel (Max EVP) assay for identification of viral pathogens in stool specimens from individuals with symptoms of acute gastroenteritis, enteritis, or colitis.

## INTRODUCTION

Molecular techniques for enteropathogen detection provide a comprehensive, rapid, and streamlined alternative to conventional methods for diagnosing microbiological causes of diarrhea. The potential advantages include improved performance parameters, a more extensive menu of pathogens, and a relatively short turnaround time (1).

Several commercial gastrointestinal multiplex PCR assays are now being widely used in clinical laboratories and target a wide range of bacterial, viral, and parasitic enteropathogens. These include the BioFire FilmArray gastrointestinal (GI) panel (bioMérieux) (1), the Luminex xTag GI pathogen panel (Luminex Corporation) (2), and the Luminex Nanosphere Verigene enteric pathogen panel (3).

At the analytical level, diagnostic stewardship interventions for limiting inappropriate testing for enteropathogens can include limiting the number of enteropathogens for which tests are conducted. Instead of performing comprehensive multiplex PCR panels, BD Max offers several multiplex PCR assays with the more selective detection of enteropathogens. These include the BD Max enteric bacterial panel (Max EBP), which detects *Salmonella*, *Shigella*, *Campylobacter*, and Shiga toxin-producing enterohemorrhagic Escherichia coli (EHEC) (4, 5); the BD Max extended enteric bacterial panel (Max xEBP), which detects Yersinia enterocolitica, enterotoxigenic Escherichia coli (ETEC), *Vibrio*, and Plesiomonas shigelloides (6); the BD Max enteric parasite panel (Max EPP), which detects Giardia lamblia, *Cryptosporidium* spp. (Cryptosporidium parvum and Cryptosporidium hominis), and Entamoeba histolytica (7, 8); and the BD Max enteric viral panel (Max EVP), which detects norovirus genogroup I (GI) and GII, rotavirus type A, adenovirus type F 40/41, human astrovirus (hAstro), and sapovirus (genogroups I, II, IV, and V).

The aim of this multisite study was to evaluate the performance of the Max EVP assay for its use in determining the presence of enteric viral pathogens from Cary-Blair medium-preserved or unpreserved stool specimens collected from individuals with symptoms of acute gastroenteritis, enteritis, or colitis.

(These data were presented at the 29th European Congress of Clinical Microbiology & Infectious Diseases, Amsterdam, Netherlands, 13 to 16 April 2019.)

## MATERIALS AND METHODS

### Specimen preparation.

Prospective specimens were included if they were from pediatric or adult patients suspected of having gastroenteritis, enteritis, or colitis. Specimens from patients in outpatient and hospital settings were included and were collected from five centers within the United States and one center in Canada between November 2016 and April 2017. Specimens were required to have a soft to diarrheal consistency and to be collected either in 15 ml of Cary-Blair medium or unpreserved in a sterile container. Specimen collection and transport were performed per standard operating procedures at each respective study site. Specimens were excluded if they had one or more of the following: were from solid or formed stools, were collected from rectal swabs, were improperly collected (e.g., specimens were unlabeled or mislabeled or specimens were in broken or leaking containers), were submitted only for Clostridioides difficile testing from patients suspected of C. difficile infection, or were collected from patients who had already tested positive for a viral enteropathogen.

Archival samples (either in Cary-Blair medium or unpreserved) were collected between November 2011 and March 2017 and used when prospective samples failed to provide an adequate number of specimens positive for specific viruses. In addition to meeting the same inclusion criteria of prospective stool specimens, archival specimens had to be positive for at least one of the following targets: norovirus, rotavirus, adenovirus, sapovirus, or astrovirus. Archival stool specimens were excluded by the same procedure used for the prospectively collected stool specimens, with the following exceptions: specimens from patients suspected of C. difficile infection or specimens not collected or transported according to each center’s standard operating procedures could be included.

Prospective specimens were split into two aliquots; one was frozen at −20 to −70°C for reference method (RM) testing; the other was tested by the Max EVP assay within 5 days from collection when it was stored at 2 to 8°C or tested within 48 h when it was stored at 2 to 25°C. Archival specimens were split into two aliquots that were stored frozen at −20 to −70°C prior to testing. One was used for RM testing and the other was used for testing with the Max EVP. For the Max EVP, specimens were vortexed and transferred into a Max sample buffer tube using a Max EVP inoculation loop (9). The Max sample buffer tube was subsequently closed with a septum cap, vortexed, and loaded onto the BD Max system along with a Max EVP unitized reagent strip and Max PCR cartridge.

### Max EVP assay.

Stool specimens were tested for enteric viral pathogens using the Max EVP on the BD Max system. The Max EVP testing was performed according to the manufacturer’s instructions (10). Proprietary probes for this assay are labeled with different fluorophores to detect norovirus GI and GII, rotavirus type A, adenovirus type F 40/41, astrovirus, and sapovirus (genogroups I, II, IV, and V). Each Max EVP assay operator was trained to handle and perform the assay at each collection site. Individuals performing the assay were blind to the results of the RM.

### Composite reference method.

Prospective and archival collected stool specimens (from frozen aliquots) were analyzed by a RM that was performed at an internal BD site in Sparks, MD, USA, between September 2017 and January 2018. The RM for prospective specimens consisted of two sets of validated, alternate PCRs (using proprietary primer sets), followed by bidirectional sequencing of the amplicons from PCR set 2 only. The composite RM was generated according to guidance provided by the Food and Drug Administration for level-of-detection testing and demonstration of analytical reactivity for gastrointestinal microorganism detection from stool specimens using multiplex nucleic acid amplification tests (11). PCR set 1 was performed using TaqMan polymerase (to enhance sensitivity) and was given greater weight in the RM algorithm. A positive result for PCR set 1 was sufficient for a positive RM result. PCR set 2 was generated with primer sets to achieve melt chemistry that optimizes specificity (Table 1). For archival specimens, the historical result as well as the results of one set of a validated alternate PCR per target and bidirectional sequencing was used. The alternate PCRs were performed for all targets for which a historical result was available. Viral nucleic acid extraction was performed using Roche MagNA Pure LC 2.0 software (version 1.1.24.1401) and a MagNA Pure LC total nucleic acid isolation kit—high performance. All real-time PCR tests were performed in simplex on a Bio-Rad CFX96 system. Touch real-time PCR detection systems (9, 12) were operated according to the instruments’ instructions with CFX manager software (version 2.0 or later) (12). Positive and negative controls were performed with every extraction and PCR run. The individuals performing the alternate PCR and sequencing were not involved in testing specimens with the BD Max EVP assay and were masked to the BD Max EVP results.

**TABLE 1:** Reference method final result adjudication algorithm for prospective and archival samples^*b*^

Sample set	Historical result (interpretation)	Result			
		PCR set 1	PCR set 2^*a*^	Sequencing on PCR set 2	Final RM result
Prospective					
		Positive	Positive	Positive	Positive
		Positive	Positive	Negative	Positive
		Negative	Positive	Positive	Positive
		Positive	Negative	Not performed	Positive
		Negative	Positive	Negative	Negative
		Negative	Negative	Not performed	Negative
Archival					
	Positive (confirmed)		Positive		Positive
	Negative (confirmed)		Negative		Negative
	Positive (unconfirmed)		Negative		NA
	Negative (unconfirmed)		Positive		NA

a Only PCR set 2 involved bidirectional sequencing for RM testing in both prospective and archival specimens.

b Abbreviations: Max, BD Max platform; RM, reference method; NA, not applicable (specimens with unconfirmed historical results were not included in the final performance calculations).

Bidirectional sequencing was performed using an Advanced Biosystems GA 3500xl genetic analyzer according to the user guide and GA3500xl data collection software (version 3.0) (13). BLAST sequence identification was performed using a proprietary SAngerSeqId validated protocol, built using the (NCBI) GenBank database (14). If the BLAST results obtained from the NCBI Databases met the acceptance criteria of a QV_20_ of ≥90% (QV is a per-base estimate of base caller accuracy, with QV_20_ implying a 1% probability of error at a given base) and an E value of ≤10e^−30^ (with a percentage of query coverage of ≥90% and a percent identity of ≥95%) for both sequence directions and the forward and reverse results were in agreement for target detection, the identification obtained was recorded as the final result and the sample was recorded as true positive. Positive and negative controls were performed with each sequencing run. Samples that had a reportable result (positive or negative) after the initial testing or upon repeat testing were included in the performance analyses, while samples that did not give a reportable result (invalid or indeterminate) after repeat testing were excluded.

### Quality control and nonreportable results.

Positive and negative controls, as well as a sample processing control (SPC), were included in each Max EVP run. The sample processing control was present in the extraction tube and subjected to lysis, extraction, concentration, and amplification steps. The SPC monitors for the presence of potential inhibitory substances as well as system or reagent failures.

A total of three repeats were allowed in this clinical study for prospective and archival specimen results that were nonreportable. Only one repeat was allowed for the RM. Nonreportable results included unresolved, indeterminate, or incomplete results. An unresolved result occurred when there was a failure of the internal control. An indeterminate result occurred when either the positive or the negative control failed, there was failure to obtain concordant results between forward and reverse sequences, and/or when not all sequencing parameters (QV_20_, percent identity, and percent query coverage) met the acceptance criteria. An incomplete result was assigned whenever an instrument error occurred. Nonevaluable samples with regard to RM testing were categorized as being either (i) due to no historical result or missing information for one or more target viruses or (ii) due to an inability to confirm the archival result with the reference method.

### Data analysis.

Based on target prevalence and experimental restraints associated with the acceptance criteria, experimental error, and other factors, the initial sample size calculation resulted in a requirement of 25 positive results each for appropriate statistical analyses related to adenovirus, sapovirus, and astrovirus; 65 positive results each were required for effective statistical analyses related to norovirus and rotavirus. An estimated enrollment minimum of 1,500 prospective specimens was deemed necessary to obtain the minimum number of positive results for each viral target. Results obtained from the prospective and archival samples were compared to the composite RM result. With these results, the positive percent agreement (PPA) and negative percent agreement (NPA) were calculated with 95% confidence intervals (CI). Prevalence rates were calculated as the number of prospective specimens that tested positive by the RM divided by the total number of prospectively enrolled specimens. Only the prospective specimens were used in the prevalence calculations. Logistic regression was performed to determine whether specimen type (preserved in Cary-Blair medium versus unpreserved), specimen class (prospective versus archival), or test site had any statistically significant impact on the PPA and NPA of the Max EVP assay with the composite RM.

## RESULTS

### Demographic data.

**FIG 1:**
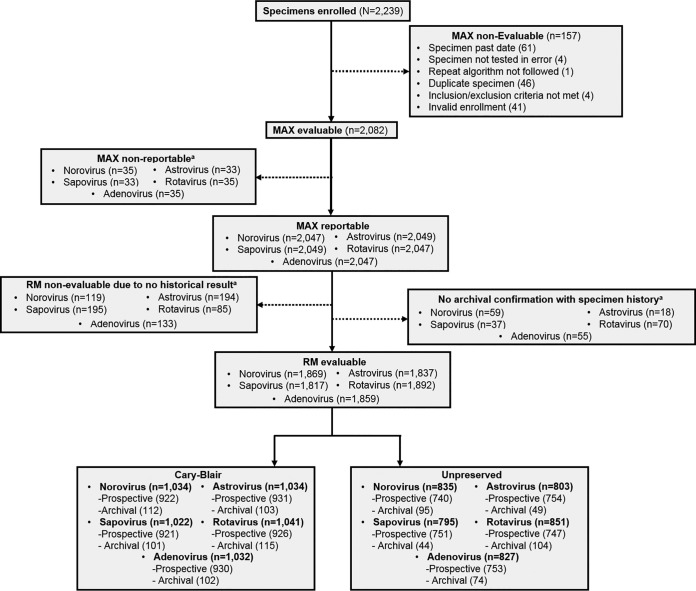
Flowchart depicting the study design. ^a^, a further description of nonreportable and nonevaluable specimens can be found in Materials and Methods.

Of the 2,239 specimens enrolled, 1,873 were prospectively collected and 366 were archival. Of the enrolled specimens, 157 were excluded due to being nonevaluable (Fig. 1; see also Fig. S1 and S6 in the supplemental material). From the remaining 2,082 specimens, 35, 33, 33, 35, and 35 specimens for norovirus, sapovirus, astrovirus, rotavirus, and adenovirus, respectively, were nonreportable for Max EVP. Of the Max EVP reportable specimens, 178, 232, 212, 155, and 188 specimens were nonevaluable by the RM for norovirus, sapovirus, astrovirus, rotavirus, and adenovirus, respectively (Fig. 1). More specimens were from patients aged >21 years (56.2%) than from those aged ≤21 years (42.1%) (Table 2). A total of 45.9% and 49.7% of specimens were collected from male and female patients, respectively. Specimens from inpatients, outpatients, emergency departments, and long-term-care facilities accounted for 37.9%, 39.6%, 10.7%, and 0.3%, respectively. A small proportion of specimens did not specify the patients’ age, gender, and/or location.

**TABLE 2:** Age group, gender, and health care setting by specimen type

Characteristic	% (no.) of subjects by specimen type		
	CB^*a*^ preserved (*n* = 1,146)	Unpreserved (*n* = 1,002)	Combined (*n* = 2,148)
Age group			
0–1 mo	0.4 (4)	0.0 (0)	0.2 (4)
1 mo–2 yr	16.4 (188)	11.2 (112)	14.0 (300)
2–12 yr	19.9 (228)	15.3 (153)	17.7 (381)
13–18 yr	10.2 (117)	6.6 (66)	8.5 (183)
19–21 yr	1.7 (20)	2.1 (21)	1.9 (41)
>21 yr	49.6 (568)	63.9 (640)	56.2 (1,208)
Unknown	1.8 (21)	1.0 (10)	1.4 (31)
Gender			
Male	44.9 (514)	47.0 (471)	45.9 (985)
Female	52.1 (597)	46.9 (470)	49.7 (1,067)
Unknown	3.1 (35)	6.1 (61)	4.5 (96)
Health care setting			
Inpatient	27.7 (318)	49.6 (497)	37.9 (815)
Outpatient	55.8 (640)	21.0 (210)	39.6 (850)
Emergency	7.6 (87)	14.3 (143)	10.7 (230)
Long-term-care facility	0.0 (0)	0.6 (6)	0.3 (6)
Unknown	8.8 (101)	14.6 (146)	11.5 (247)

a CB, Cary-Blair medium.

### Viral prevalence and Max EVP assay performance.

Of the 1,873 prospectively collected stool specimens, 1,055 came from specimens collected in Cary-Blair transport medium and 818 came from unpreserved specimens. For both specimen types combined, the overall prevalence rate for prospectively collected specimens was 7.3%, 4.5%, 3.5%, 2.4%, and 1.2% for norovirus, sapovirus, astrovirus, rotavirus, and adenovirus, respectively (Table 3). Viral enteropathogen prevalence was the highest among younger age cohorts, with the majority being detected among patients ≤12 years of age (data not shown). Performance values for individual viral targets are stratified by specimen type and collection procedure in Table 3. Max EVP achieved a ≥90% PPA with RM for 4 out of 5 viral targets; PPA values for Max EVP were the highest for rotavirus (100%; 95% CI, 97.3%, 100%) and adenovirus (95.6%; 95% CI, 85.2%, 98.8%). Max EVP achieved an 84.9% (95% CI, 75.8%, 90.9%) PPA with RM for the detection of sapovirus. The PPA value for astrovirus and norovirus was 93.0% (95% CI, 85.6, 96.8) and 92.8% (95% CI, 87.8, 95.8), respectively. Overall NPA values were ≥99.0% for all viral targets and were consistent across specimen types and specimen collection methods within each viral group.

**TABLE 3:** Performance of the Max EVP by organism, specimen type, and specimen origin^*f*^

Organism (% prevalence^*g*^) and specimen type	No. of samples with the following result:					% agreement (95% CI)	
	True positive	False positive	False negative	True negative	Total	PPA	NPA
Norovirus (7.3%)^*a*^							
Prospective							
CB preserved	74	7	6	835	922	92.5 (84.6, 96.5)	99.2 (98.3, 99.6)
Unpreserved	39	3	4	694	740	90.7 (78.4, 96.3)	99.6 (98.7, 99.9)
Archival							
CB preserved	6	1	0	105	112	100 (61.0, 100)	99.1 (94.8, 99.8)
Unpreserved	35	0	2	58	95	94.6 (82.3, 98.5)	100 (93.8, 100)
Total	154	11	12	1,692	1,869	92.8 (87.8, 95.8)	99.4 (98.8, 99.6)
Sapovirus (4.5%)^*b*^							
Prospective							
CB preserved	43	9	6	863	921	87.8 (75.8, 94.3)	99.0 (98.1, 99.5)
Unpreserved	24	1	6	720	751	80.0 (62.7, 90.5)	99.9 (99.2, 100)
Archival							
CB preserved	2	0	1	98	101	66.7 (20.8, 93.9)	100 (96.2, 100)
Unpreserved	4	1	0	39	44	100 (51.0, 100)	97.5 (87.1, 99.6)
Total	73	11	13	1,720	1,817	84.9 (75.8, 90.9)	99.4 (98.9, 99.6)
Astrovirus (3.5%)^*c*^							
Prospective							
CB preserved	29	1	2	899	931	93.5 (79.3, 98.2)	99.9 (99.4, 100)
Unpreserved	28	2	2	722	754	93.3 (78.7, 98.2)	99.7 (99.0, 99.9)
Archival							
CB preserved	20	1	2	80	103	90.9 (72.2, 97.5)	98.8 (93.3, 99.8)
Unpreserved	3	1	0	45	49	100 (43.9, 100)	97.8 (88.7, 99.6)
Total	80	5	6	1,746	1,837	93.0 (85.6, 96.8)	99.7 (93.9, 99.9)
Rotavirus (2.4%)^*d*^							
Prospective							
CB preserved	31	7	0	888	926	100 (89.0, 100)	99.2 (98.4, 99.6)
Unpreserved	11	1	0	735	747	100 (74.1, 100)	99.9 (99.2, 100)
Archival							
CB preserved	38	1	0	76	115	100 (90.8, 100)	98.7 (93.0, 99.8)
Unpreserved	56	1	0	47	104	100 (93.6, 100)	97.9 (89.1, 99.6)
Total	136	10	0	1,746	1,892	100 (97.3, 100)	99.4 (99.0, 99.7)
Adenovirus (1.2%)^*e*^							
Prospective							
CB preserved	15	0	1	914	930	93.8 (71.7, 98.9)	100 (99.6, 100)
Unpreserved	4	1	1	747	753	80.0 (37.6, 96.4)	99.9 (99.2, 100)
Archival							
CB preserved	18	0	0	84	102	100 (82.4, 100)	100 (95.6, 100)
Unpreserved	6	0	0	68	74	100 (61.0, 100)	100 (94.7, 100)
Total	43	1	2	1,813	1,859	95.6 (85.2, 98.8)	99.9 (99.7, 100)

a 127/1,751 (7.3%) specimens for norovirus.

b 80/1,760 (4.5%) specimens for sapovirus.

c 62/1,773 (3.5%) specimens for astrovirus.

d 43/1,763 (2.4%) specimens for rotavirus.

e 21/1,773 (1.2%) specimens for adenovirus.

f Abbreviations: Max EVP, BD Max enteric viral panel; PPA, positive percent agreement; NPA, negative percent agreement; CB, Cary-Blair medium.

g Prevalence values are based on detection by the reference method among the prospective specimens only.

There were a total of 71 (3.4%) discrepant results, with 24, 23, 11, 10, and 3 discrepant results being found for sapovirus, norovirus, astrovirus, rotavirus, and adenovirus, respectively. In most cases, prospective samples had higher numbers of both false-positive and false-negative results (versus the results of RM) than archival samples. In general, within viral groups there were no statistically significant differences in PPA across specimen type or specimen collection method for the performance values listed in Table 3.

There were a total of 2.8% nonreportable results during initial testing with the prospective and archival samples combined. Nonreportable results decreased to 1.0% after repeat testing (Table S13).

## DISCUSSION

Compared to other gastrointestinal multiplex PCR panels that target a full spectrum of bacterial, viral, and parasitic enteropathogens, BD Max offers a multitude of different panels that target a more selective spectrum of enteropathogens. These include the Max EBP, the Max xEBP, the Max EPP, and the Max EVP, all of which are FDA cleared. These panels can be used, selectively, to improve productivity and save costs. For instance, broader multiplex PCR panels can be restricted to at-risk patient groups, such as immunocompromised hosts, patients with relevant epidemiological risk factors, or patients with severe or more persistent symptoms. Max EVP can be added to other Max panels, or it can be used individually in certain situations where a viral enteropathogen is likely (e.g., outbreak or pediatric settings). Although gastrointestinal multiplex PCR assays require more up-front expense than conventional testing, several studies have demonstrated reduced overall costs when other factors are considered, such as additional testing, hospital length of stay, and treatment duration (15, 16). There are no cost-effectiveness studies comparing Max EVP to other multiplex PCR panels. However, the Max system is likely to further improve costs by limiting the number of PCR reagents consumed. Within the United States, it may also help increase approved medical insurance reimbursement rates, as not all medical insurance companies are willing to cover broad multiplex panels (17).

Max EVP performance was consistent and robust across the five viral targets by age, gender, and stool collection method. The performance for viral detection was comparable to that of other multiplex PCR panels (1–3). While the NPA here was ≥99.4% for all viral targets, the PPA values for norovirus, astrovirus, rotavirus, and adenovirus were 92.8%, 93.0%, 100%, and 95.6%, respectively. Sapovirus had a PPA of 84.9% in these analyses, which was lower than that found in an analysis from previous work (PPA, 100%) involving the BioFire FilmArray GI panel (bioMérieux) (1). However, preliminary head-to-head testing between Max EVP and a commercially available multiplex assay (FilmArray GI panel) revealed high concordance relative to RM (data not shown). Additional head-to-head testing is required to confirm these results.

There were a total of 71 discrepant results, with the majority occurring among samples positive for sapovirus and/or norovirus. While it appears that the false positives and false negatives occurred more commonly among the prospective samples, these differences were not statistically significant, with the exception of astrovirus, for which a difference in NPA between the prospective and archival results was detected (*P* = 0.019; data not shown). This slight difference in NPA might have been due to the low number of archival samples tested. Although prospective collection is ideal for diagnostic studies, such as the one described here, results from archival specimens were included in the data analyses. However, inclusion of archival specimens was done here according to guidance provided by the FDA (11) and was necessary for statistical analyses and to meet confidence interval limit and range values. In addition, this study was performed in a blind manner, and the technicians that performed the Max EVP assay had no knowledge of the reference method results for archival or prospective samples; similarly, the technicians performing reference method testing had no knowledge of the Max EVP results. Therefore, we do not believe that the archival nature of the samples had a significant impact on the performance values that would fundamentally differentiate them from prospective samples during the conduct of these experiments.

Interestingly, many discrepant results (24/71) were from samples with multiple viral enteropathogens detected by either the Max EVP or the RM. Out of the 24 discrepant results, 19 were due to false-positive results with the Max EVP. However, similar false-positivity rates have previously been associated with coinfections on other platforms using molecular detection of enteropathogens (18). The cause(s) for this association is not clear, and further research is required to definitively establish this finding. To minimize optical cross talk, the BD Max five-channel fluorescence reader design includes excitation and emission filters, independent channel excitation and detection, narrow-band spectral filters, and integrated compensation factors. However, it is still possible that false positives occur as a result of channel bleed-through during sample detection on the BD Max platform.

This study was a large, multisite study that analyzed the Max EVP for viral enteropathogen detection among symptomatic patients across a wide variety of age groups, patient locations, and geographic areas. This study included a large number of specimens positive for viral enteropathogens. For instance, this study included 166, 136, and 86 samples positive for norovirus, rotavirus, and astrovirus, respectively. Here, pediatric patients (some <1 month old) and adult patients from various areas within the United States and Canada were included. The Max EVP was tested with specimens from various patient settings, including inpatient, outpatient, emergency room, and long-term-care facilities. Furthermore, these data suggest that specimens preserved in Cary-Blair medium and unpreserved stool specimens can be used with similar efficacy for testing on the Max EVP.

Noncompliant (invalid, unresolved) results within this study were minimal, representing 2.8% of the total rate, which decreased to 0.6% when the sample was repeat tested with the Max EVP (up to three times). Other BD Max gastrointestinal panels had similar noncompliance rates (5, 6), which are comparable to those for other gastrointestinal multiplex PCR panels (1).

While the sample size was large, it could not reliably provide PPA/NPA data for every virus when split between specimen type and each individual age group. Furthermore, the clinical relevance of detecting viral DNA/RNA in clinical specimens is still in question. Detecting viral DNA/RNA may lead to a false diagnosis, as was observed in a case-control study from Denmark which detected enteropathogenic viruses using PCR in 3% of healthy controls (19). The prevalence of viral enteropathogens among healthy hosts is higher among developing countries as a result of improper access to sanitary water. One study in China, for instance, detected a viral enteropathogen in 47.5% of hospitalized children without diarrhea using PCR-based methods (20). This drawback is not limited to viral etiologies, however, as similar results have been seen for many enteropathogens detected using PCR-based methods (21). In many situations, incorporation of molecular-based tests for viral causes of enteric disease should not be relied on solely for a diagnosis; rather, they should be included as part of a diagnostic algorithm that includes a patient interview and a review of symptoms, the history of travel, and other pertinent clinical factors to achieve a proper diagnosis. In our study, we included only patients with symptoms and signs consistent with gastroenteritis, thereby increasing the pretest probability and limiting the number of clinically irrelevant positive results. Preanalytic interventions can improve the pretest probability when multiplex molecular panels are used. Such interventions include the use of case history forms, risk stratification, diagnostic algorithms to guide testing, renewed training, and education for health care providers regarding the appropriate application of syndromic testing panels and modifications to hospital work flows (22).

In conclusion, Max EVP reliably detects the presence or absence of viral enteropathogens. It can be used individually as a selective diagnostic test for patients at high risk for a viral enteropathogen or as an additive assay to other BD Max panels, such as the Max enteric bacterial panels. It allows targeted testing for enteropathogens in patients presenting with gastroenteritis, in concert with other selective BD Max enteric panels.

## Supplementary Material

Supplemental file 1
