# Supplementary material for: Multicenter Clinical Validation of the Molecular BD Max Enteric Viral Panel for Detection of Enteric Pathogens
Source: J Clin Microbiol. 2019 Aug 26;57(9):e00306-19. doi: 10.1128/JCM.00306-19 (PMC6711915; doi:10.1128/JCM.00306-19)
Supplement: Supplemental file 1 [file JCM.00306-19-s0001.pdf]

Table of Contents

Figure 1: Compliance flow chart – prevalence data set	3
Figure 2: Compliance Flow Chart for Norovirus	4
Figure 3. Compliance Flow Chart for Rotavirus	5
Figure 4. Compliance Flow Chart for Adenovirus	6
Figure 5. Compliance Flow Chart for Sapovirus	7
Figure 6. Compliance Flow Chart for Astrovirus	8
Table 1: Reasons for Specimen Non Compliance per Site for Norovirus	9
Table 2: Specimen Types by Sites (Prospective Collection) and States/Provinces	9
Table 3: PPA and NPA Summary for all Targets by Specimen Types and Origin	10
Table 4: PPA and NPA Summary for all Targets by Age group	11
Table 5: NPA and PPA Summary for all Targets by Patient Population	15
Table 6: Poolability	18
Table 7: Non-Reportable Rate for Combined Target by Specimen Type, Site and Overall	19
Table 8: Discrepant Norovirus Results	19
Table 9: Discrepant Rotavirus Results	20
Table 10: Discrepant Astrovirus Results	20
Table 11: Discrepant Sapovirus Results	21
Table 12: Discrepant Adenovirus Results	22
Appendix A: Further details on the reference method	23
Table A1. Non-sequencing reference method assays	27
Table A2. Summary of Reference Method Validation Results	28
Table A3. Alternative PCR inclusivity—non-sequencing assay results	29
Table A4. Alternative PCR inclusivity—sequencing assay results	30

Appendix B: Repeat strategy 31
Table B1. Total Non-Reportable Rate for Combined Target by Specimen Type and Overall (BD MAX EVP) 31

Figure 1. Compliance Flow Chart – Prevalence Data Set

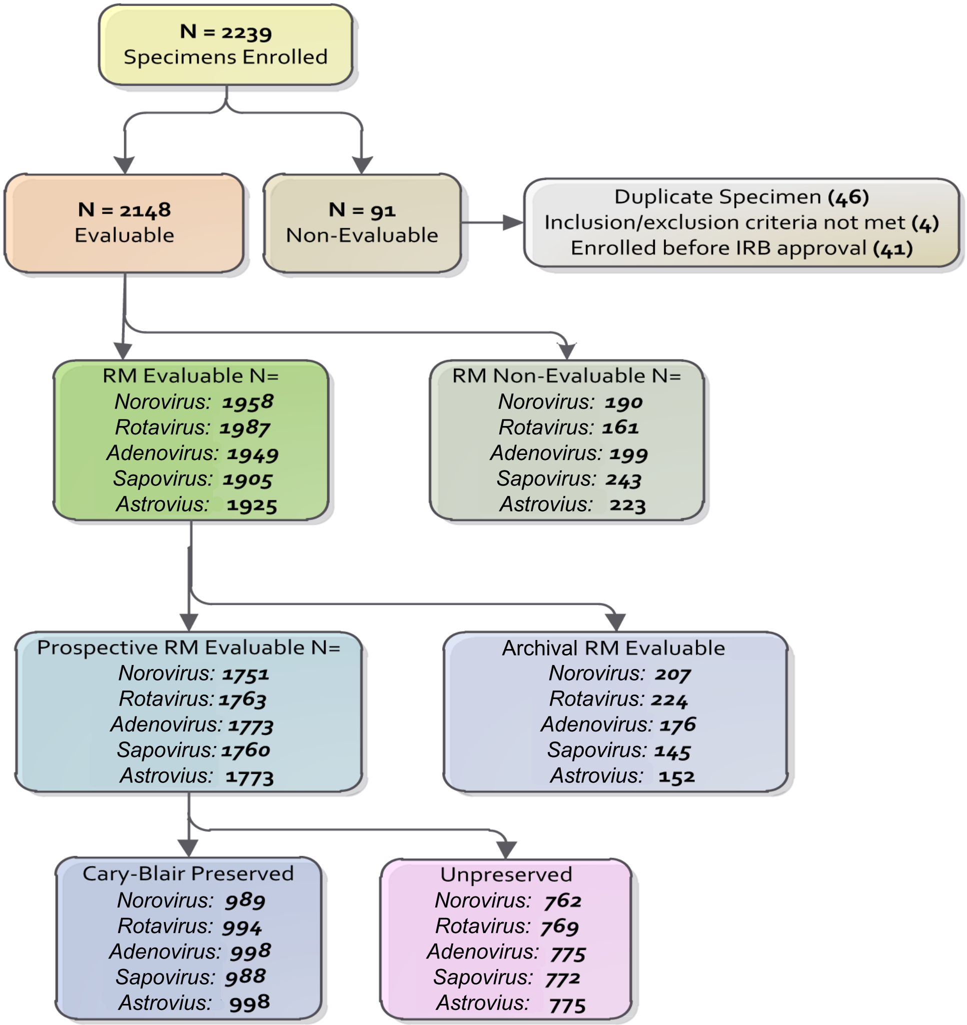

RM: reference method

Figure 2. Compliance Flow Chart for Norovirus

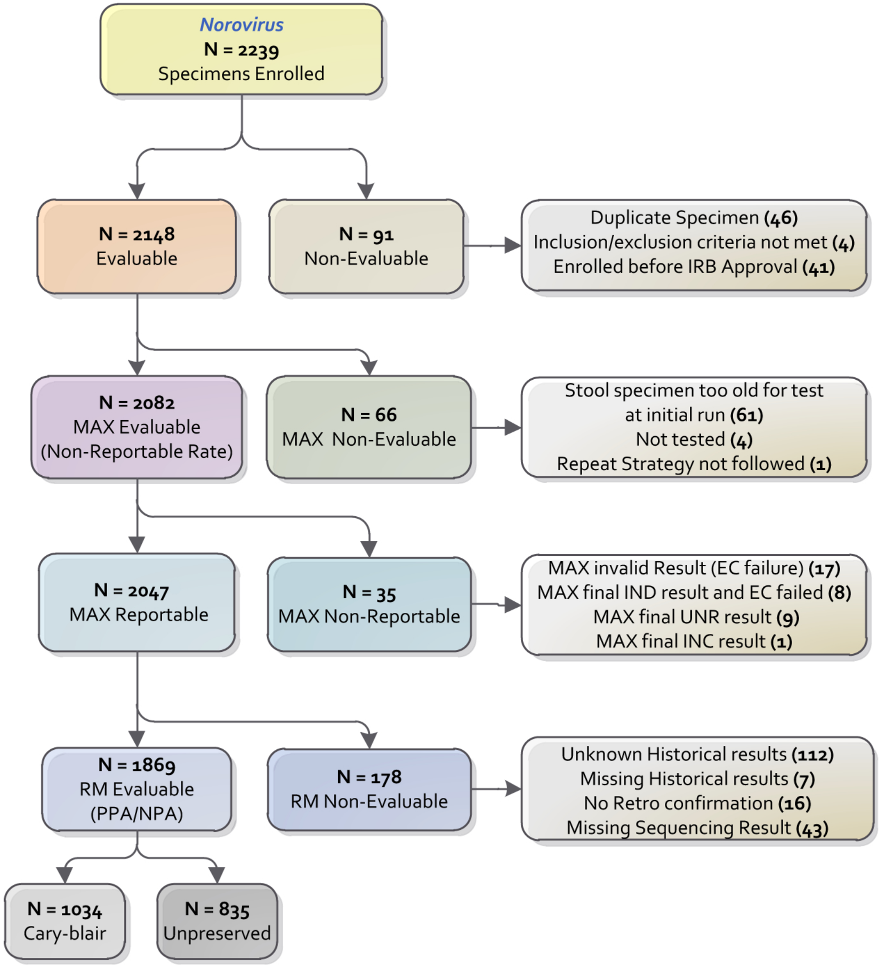

RM: reference method

Figure 3. Compliance Flow Chart for Rotavirus

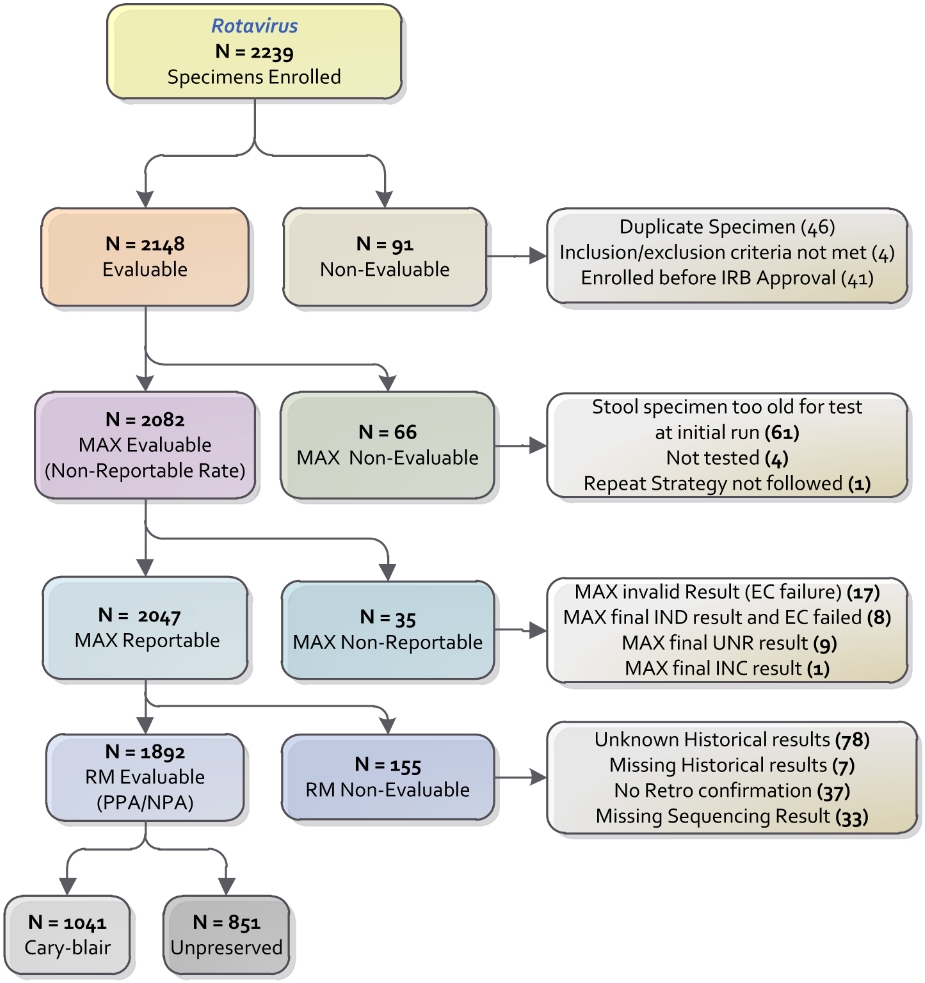

RM: reference method

Figure 4. Compliance Flow Chart for Adenovirus

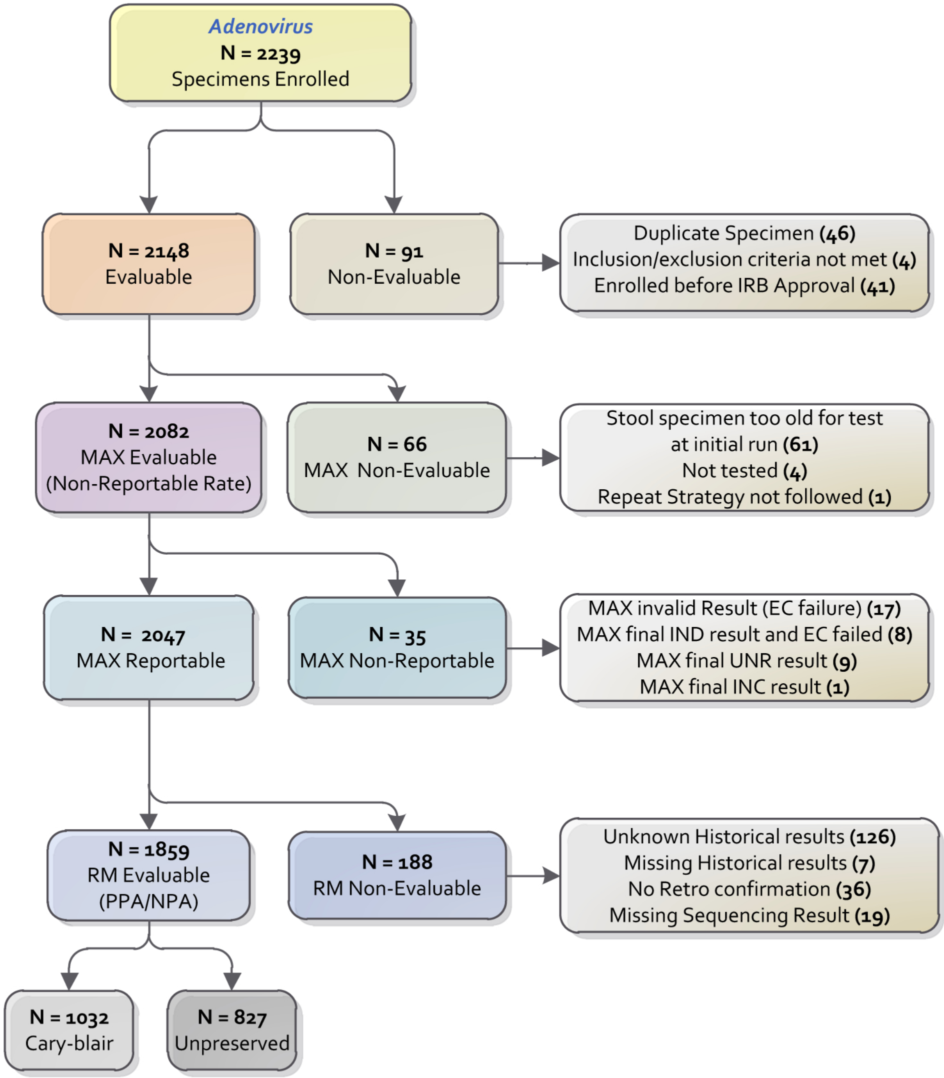

RM: reference method

Figure 5. Compliance Flow Chart for Sapovirus

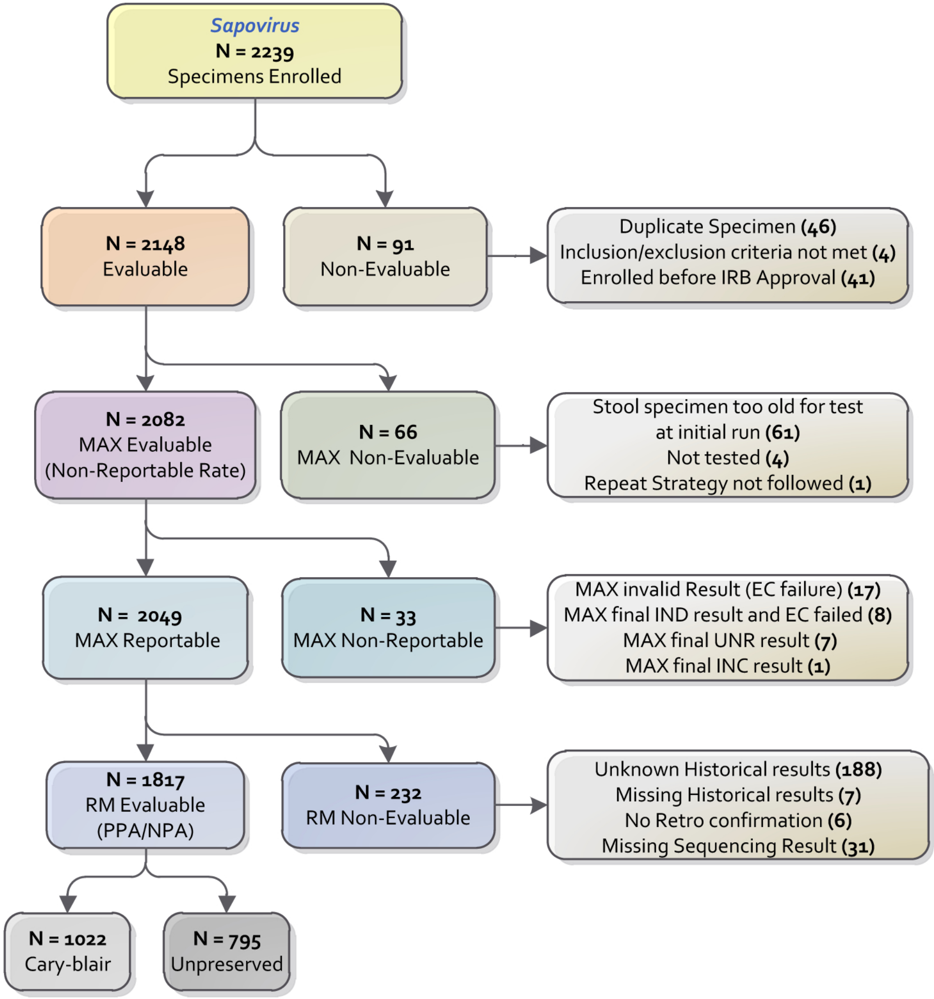

RM: reference method

Figure 6. Compliance Flow Chart for Astrovirus

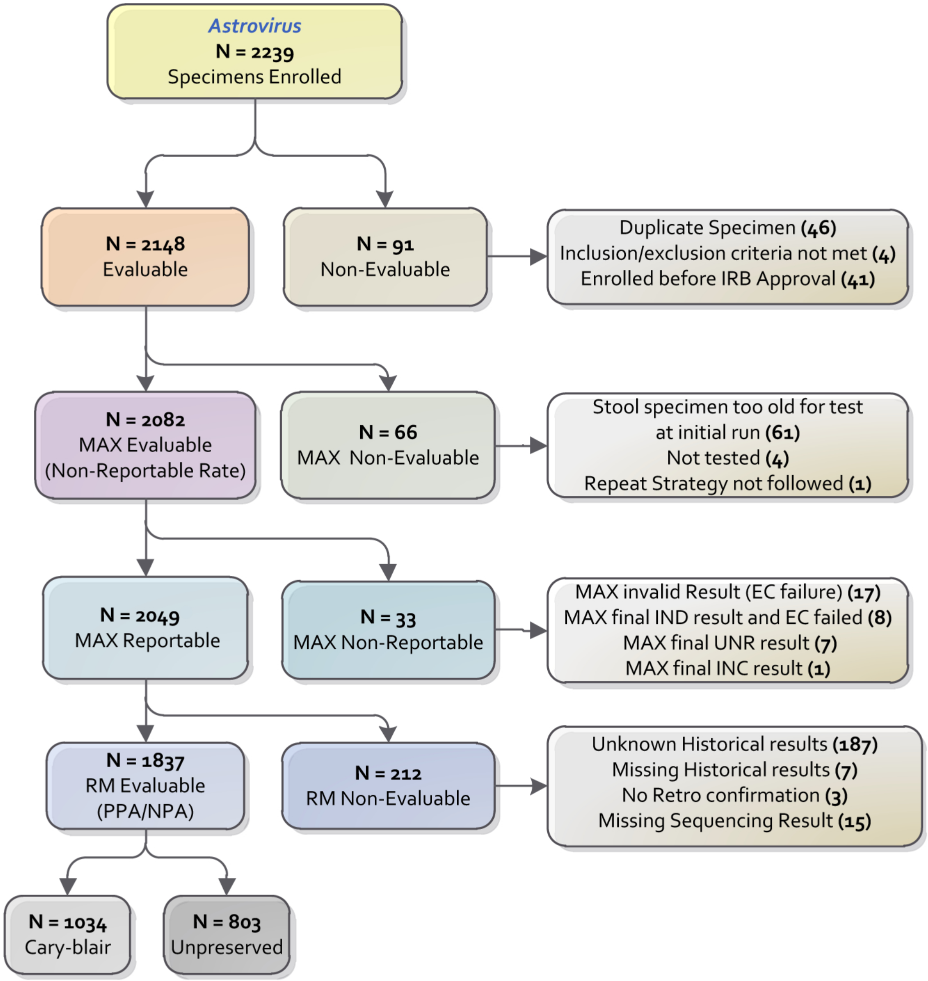

RM: reference method

Table 1: Reasons for Specimen Non Compliance for all targets

		Virus				
**Compliant**	**Description**	**Norovirus**	**Rotavirus**	**Adenovirus**	**Sapovirus**	**Astrovirus**
No	BD MAX: No Data	3	3	3	3	3
	BD MAX: Repeat strategy not followed properly	1	1	1	1	1
	BD MAX: Specimen too old for testing - Collection vs SBT	55	56	56	56	56
	Composite Reference Method: No Data	183	155	193	237	217
	Composite Reference Method: No Data/BD MAX: No Data	1	1	1	1	1
	Composite Reference Method: No Data/BD MAX: Specimen too old for testing - Collection vs SBT	6	5	5	5	5
	Enrolled before IRB renewal	41	41	41	41	41
	Inclusion/Exclusion criteria not respected	4	4	4	4	4
Yes	More than one Specimen enrolled for the same patient	46	46	46	46	46
	Fully Compliant	1899	1927	1889	1845	1865
Total		2239	2239	2239	2239	2239

Table 2: Specimen Types by Sites (Prospective Collection) and States/Provinces

	All Specimens		
Site and State	***Cary-Blair Preserved***	***Unpreserved***	***Combined***
CIN (Ohio)	395	3	398
LUR (Illinois)	0	104	104
JHH (Maryland)	185	69	254
POR (Oregon)	410	13	423
ALB (New York)	65	1	66
CAL (Alberta)	0	628	628
Total	1055	818	1873

Table 3: PPA and NPA Summary for all Targets by Specimen Types and Origin

		Norovirus		Rotavirus		Adenovirus		Sapovirus		Astrovirus	
		**PPA**	**NPA**	**PPA**	**NPA**	**PPA**	**NPA**	**PPA**	**NPA**	**PPA**	**NPA**
**Specimen Type**	**Specimen Origin**	**(95% CI)**	**(95% CI)**	**(95% CI)**	**(95% CI)**	**(95% CI)**	**(95% CI)**	**(95% CI)**	**(95% CI)**	**(95% CI)**	**(95% CI)**
Cary-Blair Preserved	Prospective	92.5%	99.2%	100.0%	99.2%	93.8%	100.0%	87.8%	99.0%	93.5%	99.9%
		74/80	835/842	31/31	888/895	15/16	914/914	43/49	863/872	29/31	899/900
		(84.6%, 96.5%)	(98.3%, 99.6%)	(89.0%, 100.0%)	(98.4%, 99.6%)	(71.7%, 98.9%)	(99.6%, 100.0%)	(75.8%, 94.3%)	(98.1%, 99.5%)	(79.3%, 98.2%)	(99.4%, 100.0%)
	Retrospective	100.0%	99.1%	100.0%	98.7%	100.0%	100.0%	66.7%	100.0%	90.9%	98.8%
		6/6	105/106	38/38	76/77	18/18	84/84	2/3	98/98	20/22	80/81
		(61.0%, 100.0%)	(94.8%, 99.8%)	(90.8%, 100.0%)	(93.0%, 99.8%)	(82.4%, 100.0%)	(95.6%, 100.0%)	(20.8%, 93.9%)	(96.2%, 100.0%)	(72.2%, 97.5%)	(93.3%, 99.8%)
	Total	93.0%	99.2%	100.0%	99.2%	97.1%	100.0%	86.5%	99.1%	92.5%	99.8%
		80/86	940/948	69/69	964/972	33/34	998/998	45/52	961/970	49/53	979/981
		(85.6%, 96.8%)	(98.3%, 99.6%)	(94.7%, 100.0%)	(98.4%, 99.6%)	(85.1%, 99.5%)	(99.6%, 100.0%)	(74.7%, 93.3%)	(98.2%, 99.5%)	(82.1%, 97.0%)	(99.3%, 99.9%)
Unpreserved	Prospective	90.7%	99.6%	100.0%	99.9%	80.0%	99.9%	80.0%	99.9%	93.3%	99.7%
		39/43	694/697	11/11	735/736	4/5	747/748	24/30	720/721	28/30	722/724
		(78.4%, 96.3%)	(98.7%, 99.9%)	(74.1%, 100.0%)	(99.2%, 100.0%)	(37.6%, 96.4%)	(99.2%, 100.0%)	(62.7%, 90.5%)	(99.2%, 100.0%)	(78.7%, 98.2%)	(99.0%, 99.9%)
	Retrospective	94.6%	100.0%	100.0%	97.9%	100.0%	100.0%	100.0%	97.5%	100.0%	97.8%
		35/37	58/58	56/56	47/48	6/6	68/68	4/4	39/40	3/3	45/46
		(82.3%, 98.5%)	(93.8%, 100.0%)	(93.6%, 100.0%)	(89.1%, 99.6%)	(61.0%, 100.0%)	(94.7%, 100.0%)	(51.0%, 100.0%)	(87.1%, 99.6%)	(43.9%, 100.0%)	(88.7%, 99.6%)
	Total	92.5%	99.6%	100.0%	99.7%	90.9%	99.9%	82.4%	99.7%	93.9%	99.6%
		74/80	752/755	67/67	782/784	10/11	815/816	28/34	759/761	31/33	767/770
		(84.6%, 96.5%)	(98.8%, 99.9%)	(94.6%, 100.0%)	(99.1%, 99.9%)	(62.3%, 98.4%)	(99.3%, 100.0%)	(66.5%, 91.7%)	(99.0%, 99.9%)	(80.4%, 98.3%)	(98.9%, 99.9%)
Combined	Prospective	91.9%	99.4%	100.0%	99.5%	90.5%	99.9%	84.8%	99.4%	93.4%	99.8%
		113/123	1529/1539	42/42	1623/1631	19/21	1661/1662	67/79	1583/1593	57/61	1621/1624
		(85.7%, 95.5%)	(98.8%, 99.6%)	(91.6%, 100.0%)	(99.0%, 99.8%)	(71.1%, 97.3%)	(99.7%, 100.0%)	(75.3%, 91.1%)	(98.8%, 99.7%)	(84.3%, 97.4%)	(99.5%, 99.9%)
	Retrospective	95.3%	99.4%	100.0%	98.4%	100.0%	100.0%	85.7%	99.3%	92.0%	98.4%

		Norovirus		Rotavirus		Adenovirus		Sapovirus		Astrovirus	
		**PPA**	**NPA**	**PPA**	**NPA**	**PPA**	**NPA**	**PPA**	**NPA**	**PPA**	**NPA**
**Specimen Type**	**Specimen Origin**	**(95% CI)**	**(95% CI)**	**(95% CI)**	**(95% CI)**	**(95% CI)**	**(95% CI)**	**(95% CI)**	**(95% CI)**	**(95% CI)**	**(95% CI)**
		41/43	163/164	94/94	123/125	24/24	152/152	6/7	137/138	23/25	125/127
		(84.5%, 98.7%)	(96.6%, 99.9%)	(96.1%, 100.0%)	(94.4%, 99.6%)	(86.2%, 100.0%)	(97.5%, 100.0%)	(48.7%, 97.4%)	(96.0%, 99.9%)	(75.0%, 97.8%)	(94.4%, 99.6%)
	Total	92.8%	99.4%	100.0%	99.4%	95.6%	99.9%	84.9%	99.4%	93.0%	99.7%
		154/166	1692/1703	136/136	1746/1756	43/45	1813/1814	73/86	1720/1731	80/86	1746/1751
		(87.8%, 95.8%)	(98.8%, 99.6%)	(97.3%, 100.0%)	(99.0%, 99.7%)	(85.2%, 98.8%)	(99.7%, 100.0%)	(75.8%, 90.9%)	(98.9%, 99.6%)	(85.6%, 96.8%)	(99.3%, 99.9%)

Table 4: PPA and NPA Summary for all Targets by Age group

		Norovirus		Rotavirus		Adenovirus		Sapovirus		Astrovirus	
		PPA	NPA	PPA	NPA	PPA	NPA	PPA	NPA	PPA	NPA
Specimen Type	Age Group	(95% CI)	(95% CI)	(95% CI)	(95% CI)	(95% CI)	(95% CI)	(95% CI)	(95% CI)	(95% CI)	(95% CI)
Cary-Blair Preserved	0-1 month	No data for PPA calculation	100.0%	No data for PPA calculation	100.0%	No data for PPA calculation	100.0%	100.0%	50.0%	100.0%	100.0%
			3/3		3/3		3/3	1/1	1/2	1/1	2/2
			(43.9%, 100.0%)		(43.9%, 100.0%)		(43.9%, 100.0%)	(20.7%, 100.0%)	(9.5%, 90.5%)	(20.7%, 100.0%)	(34.2%, 100.0%)
	1 month to 2 years	97.1%	97.3%	100.0%	96.0%	92.3%	100.0%	100.0%	98.0%	90.9%	99.4%
		33/34	144/148	26/26	144/150	12/13	170/170	26/26	149/152	20/22	160/161
		(85.1%, 99.5%)	(93.3%, 98.9%)	(87.1%, 100.0%)	(91.5%, 98.2%)	(66.7%, 98.6%)	(97.8%, 100.0%)	(87.1%, 100.0%)	(94.4%, 99.3%)	(72.2%, 97.5%)	(96.6%, 99.9%)
	2-12	91.7%	99.5%	100.0%	99.5%	100.0%	100.0%	66.7%	98.5%	90.5%	100.0%
		22/24	189/190	14/14	203/204	13/13	205/205	10/15	196/199	19/21	199/199
		(74.2%, 97.7%)	(97.1%, 99.9%)	(78.5%, 100.0%)	(97.3%, 99.9%)	(77.2%, 100.0%)	(98.2%, 100.0%)	(41.7%, 84.8%)	(95.7%, 99.5%)	(71.1%, 97.3%)	(98.1%, 100.0%)
	13-18	80.0%	100.0%	100.0%	100.0%	100.0%	100.0%	100.0%	98.2%	100.0%	99.1%
		4/5	107/107	3/3	111/111	3/3	111/111	4/4	107/109	2/2	110/111
		(37.6%, 96.4%)	(96.5%, 100.0%)	(43.9%, 100.0%)	(96.7%, 100.0%)	(43.9%, 100.0%)	(96.7%, 100.0%)	(51.0%, 100.0%)	(93.6%, 99.5%)	(34.2%, 100.0%)	(95.1%, 99.8%)
	19-21	100.0%	100.0%	No data for PPA calculation	100.0%	No data for PPA calculation	100.0%	No data for PPA calculation	100.0%	100.0%	100.0%
		2/2	17/17		19/19		19/19		19/19	1/1	18/18
		(34.2%, 100.0%)	(81.6%, 100.0%)		(83.2%, 100.0%)		(83.2%, 100.0%)		(83.2%, 100.0%)	(20.7%, 100.0%)	(82.4%, 100.0%)
	Over 21	90.5%	99.4%	100.0%	100.0%	100.0%	100.0%	66.7%	100.0%	100.0%	100.0%
		19/21	475/478	18/18	479/479	4/4	483/483	4/6	482/482	5/5	484/484
		(71.1%, 97.3%)	(98.2%, 99.8%)	(82.4%, 100.0%)	(99.2%, 100.0%)	(51.0%, 100.0%)	(99.2%, 100.0%)	(30.0%, 90.3%)	(99.2%, 100.0%)	(56.6%, 100.0%)	(99.2%, 100.0%)
	Unknown	No data for PPA calculation	100.0%	100.0%	83.3%	100.0%	100.0%	No data for PPA calculation	100.0%	100.0%	100.0%
			5/5	8/8	5/6	1/1	7/7		7/7	1/1	6/6
			(56.6%, 100.0%)	(67.6%, 100.0%)	(43.6%, 97.0%)	(20.7%, 100.0%)	(64.6%, 100.0%)		(64.6%, 100.0%)	(20.7%, 100.0%)	(61.0%, 100.0%)
	Total	93.0%	99.2%	100.0%	99.2%	97.1%	100.0%	86.5%	99.1%	92.5%	99.8%
		80/86	940/948	69/69	964/972	33/34	998/998	45/52	961/970	49/53	979/981

		Norovirus		Rotavirus		Adenovirus		Sapovirus		Astrovirus	
		PPA	NPA	PPA	NPA	PPA	NPA	PPA	NPA	PPA	NPA
**Specimen Type**	**Age Group**	**(95% CI)**	**(95% CI)**	**(95% CI)**	**(95% CI)**	**(95% CI)**	**(95% CI)**	**(95% CI)**	**(95% CI)**	**(95% CI)**	**(95% CI)**
		(85.6%, 96.8%)	(98.3%, 99.6%)	(94.7%, 100.0%)	(98.4%, 99.6%)	(85.1%, 99.5%)	(99.6%, 100.0%)	(74.7%, 93.3%)	(98.2%, 99.5%)	(82.1%, 97.0%)	(99.3%, 99.9%)
Unpreserved	1 month to 2 years	84.6%	100.0%	100.0%	100.0%	100.0%	100.0%	100.0%	100.0%	No data for PPA calculation	100.0%
		11/13	42/42	30/30	35/35	3/3	61/61	6/6	38/38		46/46
		(57.8%, 95.7%)	(91.6%, 100.0%)	(88.6%, 100.0%)	(90.1%, 100.0%)	(43.9%, 100.0%)	(94.1%, 100.0%)	(61.0%, 100.0%)	(90.8%, 100.0%)		(92.3%, 100.0%)
	2-12	100.0%	98.9%	100.0%	98.9%	100.0%	100.0%	100.0%	97.7%	100.0%	97.8%
		18/18	87/88	14/14	92/93	4/4	97/97	6/6	84/86	6/6	87/89
		(82.4%, 100.0%)	(93.8%, 99.8%)	(78.5%, 100.0%)	(94.2%, 99.8%)	(51.0%, 100.0%)	(96.2%, 100.0%)	(61.0%, 100.0%)	(91.9%, 99.4%)	(61.0%, 100.0%)	(92.2%, 99.4%)
	13-18	100.0%	100.0%	100.0%	98.1%	No data for PPA calculation	100.0%	0.0%	100.0%	50.0%	100.0%
		7/7	43/43	1/1	51/52		52/52	0/2	50/50	1/2	52/52
		(64.6%, 100.0%)	(91.8%, 100.0%)	(20.7%, 100.0%)	(89.9%, 99.7%)		(93.1%, 100.0%)	(0.0%, 65.8%)	(92.9%, 100.0%)	(9.5%, 90.5%)	(93.1%, 100.0%)
	19-21	100.0%	100.0%	No data for PPA calculation	100.0%	No data for PPA calculation	100.0%	No data for PPA calculation	100.0%	No data for PPA calculation	100.0%
		6/6	13/13		14/14		14/14		14/14		14/14
		(61.0%, 100.0%)	(77.2%, 100.0%)		(78.5%, 100.0%)		(78.5%, 100.0%)		(78.5%, 100.0%)		(78.5%, 100.0%)
	Over 21	88.9%	99.6%	100.0%	100.0%	75.0%	99.8%	80.0%	100.0%	96.0%	99.8%
		32/36	557/559	15/15	589/589	3/4	590/591	16/20	572/572	24/25	567/568
		(74.7%, 95.6%)	(98.7%, 99.9%)	(79.6%, 100.0%)	(99.4%, 100.0%)	(30.1%, 95.4%)	(99.0%, 100.0%)	(58.4%, 91.9%)	(99.3%, 100.0%)	(80.5%, 99.3%)	(99.0%, 100.0%)
	Unknown	No data for PPA calculation	100.0%	100.0%	100.0%	No data for PPA calculation	100.0%	No data for PPA calculation	100.0%	No data for PPA calculation	100.0%
			10/10	7/7	1/1		1/1		1/1		1/1
			(72.2%, 100.0%)	(64.6%, 100.0%)	(20.7%, 100.0%)		(20.7%, 100.0%)		(20.7%, 100.0%)		(20.7%, 100.0%)
	Total	92.5%	99.6%	100.0%	99.7%	90.9%	99.9%	82.4%	99.7%	93.9%	99.6%
		74/80	752/755	67/67	782/784	10/11	815/816	28/34	759/761	31/33	767/770
		(84.6%, 96.5%)	(98.8%, 99.9%)	(94.6%, 100.0%)	(99.1%, 99.9%)	(62.3%, 98.4%)	(99.3%, 100.0%)	(66.5%, 91.7%)	(99.0%, 99.9%)	(80.4%, 98.3%)	(98.9%, 99.9%)
Combined	0-1 month	No data for PPA calculation	100.0%	No data for PPA calculation	100.0%	No data for PPA calculation	100.0%	100.0%	50.0%	100.0%	100.0%
			3/3		3/3		3/3	1/1	1/2	1/1	2/2

		Norovirus		Rotavirus		Adenovirus		Sapovirus		Astrovirus	
		PPA	NPA	PPA	NPA	PPA	NPA	PPA	NPA	PPA	NPA
Specimen Type	Age Group	(95% CI)	(95% CI)	(95% CI)	(95% CI)	(95% CI)	(95% CI)	(95% CI)	(95% CI)	(95% CI)	(95% CI)
			(43.9%, 100.0%)		(43.9%, 100.0%)		(43.9%, 100.0%)	(20.7%, 100.0%)	(9.5%, 90.5%)	(20.7%, 100.0%)	(34.2%, 100.0%)
	1 month to 2 years	93.6%	97.9%	100.0%	96.8%	93.8%	100.0%	100.0%	98.4%	90.9%	99.5%
		44/47	186/190	56/56	179/185	15/16	231/231	32/32	187/190	20/22	206/207
		(82.8%, 97.8%)	(94.7%, 99.2%)	(93.6%, 100.0%)	(93.1%, 98.5%)	(71.7%, 98.9%)	(98.4%, 100.0%)	(89.3%, 100.0%)	(95.5%, 99.5%)	(72.2%, 97.5%)	(97.3%, 99.9%)
	2-12	95.2%	99.3%	100.0%	99.3%	100.0%	100.0%	76.2%	98.2%	92.6%	99.3%
		40/42	276/278	28/28	295/297	17/17	302/302	16/21	280/285	25/27	286/288
		(84.2%, 98.7%)	(97.4%, 99.8%)	(87.9%, 100.0%)	(97.6%, 99.8%)	(81.6%, 100.0%)	(98.7%, 100.0%)	(54.9%, 89.4%)	(96.0%, 99.2%)	(76.6%, 97.9%)	(97.5%, 99.8%)
	13-18	91.7%	100.0%	100.0%	99.4%	100.0%	100.0%	66.7%	98.7%	75.0%	99.4%
		11/12	150/150	4/4	162/163	3/3	163/163	4/6	157/159	3/4	162/163
		(64.6%, 98.5%)	(97.5%, 100.0%)	(51.0%, 100.0%)	(96.6%, 99.9%)	(43.9%, 100.0%)	(97.7%, 100.0%)	(30.0%, 90.3%)	(95.5%, 99.7%)	(30.1%, 95.4%)	(96.6%, 99.9%)
	19-21	100.0%	100.0%	No data for PPA calculation	100.0%	No data for PPA calculation	100.0%	No data for PPA calculation	100.0%	100.0%	100.0%
		8/8	30/30		33/33		33/33		33/33	1/1	32/32
		(67.6%, 100.0%)	(88.6%, 100.0%)		(89.6%, 100.0%)		(89.6%, 100.0%)		(89.6%, 100.0%)	(20.7%, 100.0%)	(89.3%, 100.0%)
	Over 21	89.5%	99.5%	100.0%	100.0%	87.5%	99.9%	76.9%	100.0%	96.7%	99.9%
		51/57	1032/1037	33/33	1068/1068	7/8	1073/1074	20/26	1054/1054	29/30	1051/1052
		(78.9%, 95.1%)	(98.9%, 99.8%)	(89.6%, 100.0%)	(99.6%, 100.0%)	(52.9%, 97.8%)	(99.5%, 100.0%)	(57.9%, 89.0%)	(99.6%, 100.0%)	(83.3%, 99.4%)	(99.5%, 100.0%)
	Unknown	No data for PPA calculation	100.0%	100.0%	85.7%	100.0%	100.0%	No data for PPA calculation	100.0%	100.0%	100.0%
			15/15	15/15	6/7	1/1	8/8		8/8	1/1	7/7
			(79.6%, 100.0%)	(79.6%, 100.0%)	(48.7%, 97.4%)	(20.7%, 100.0%)	(67.6%, 100.0%)		(67.6%, 100.0%)	(20.7%, 100.0%)	(64.6%, 100.0%)
	Total	92.8%	99.4%	100.0%	99.4%	95.6%	99.9%	84.9%	99.4%	93.0%	99.7%
		154/166	1692/1703	136/136	1746/1756	43/45	1813/1814	73/86	1720/1731	80/86	1746/1751
		(87.8%, 95.8%)	(98.8%, 99.6%)	(97.3%, 100.0%)	(99.0%, 99.7%)	(85.2%, 98.8%)	(99.7%, 100.0%)	(75.8%, 90.9%)	(98.9%, 99.6%)	(85.6%, 96.8%)	(99.3%, 99.9%)

Table 5: NPA and PPA Summary for all Targets by Patient Population

		Norovirus		Rotavirus		Adenovirus		Sapovirus		Astrovirus	
		PPA	NPA	PPA	NPA	PPA	NPA	PPA	NPA	PPA	NPA
Specimen Type	Patient Population	(95% CI)	(95% CI)	(95% CI)	(95% CI)	(95% CI)	(95% CI)	(95% CI)	(95% CI)	(95% CI)	(95% CI)
Cary-Blair Preserved	In-patient	95.8%	99.2%	100.0%	100.0%	66.7%	100.0%	88.9%	99.2%	100.0%	99.6%
		23/24	234/236	9/9	254/254	2/3	259/259	8/9	252/254	9/9	253/254
		(79.8%, 99.3%)	(97.0%, 99.8%)	(70.1%, 100.0%)	(98.5%, 100.0%)	(20.8%, 93.9%)	(98.5%, 100.0%)	(56.5%, 98.0%)	(97.2%, 99.8%)	(70.1%, 100.0%)	(97.8%, 99.9%)
	Out-patient	91.3%	99.3%	100.0%	98.8%	100.0%	100.0%	85.4%	98.9%	91.7%	99.8%
		42/46	565/569	18/18	589/596	19/19	600/600	35/41	561/567	33/36	583/584
		(79.7%, 96.6%)	(98.2%, 99.7%)	(82.4%, 100.0%)	(97.6%, 99.4%)	(83.2%, 100.0%)	(99.4%, 100.0%)	(71.6%, 93.1%)	(97.7%, 99.5%)	(78.2%, 97.1%)	(99.0%, 100.0%)
	Emergency	92.3%	98.5%	100.0%	100.0%	100.0%	100.0%	100.0%	98.7%	66.7%	100.0%
		12/13	65/66	4/4	75/75	1/1	78/78	1/1	76/77	2/3	76/76
		(66.7%, 98.6%)	(91.9%, 99.7%)	(51.0%, 100.0%)	(95.1%, 100.0%)	(20.7%, 100.0%)	(95.3%, 100.0%)	(20.7%, 100.0%)	(93.0%, 99.8%)	(20.8%, 93.9%)	(95.2%, 100.0%)
	Unknown	100.0%	98.7%	100.0%	97.9%	100.0%	100.0%	100.0%	100.0%	100.0%	100.0%
		3/3	76/77	38/38	46/47	11/11	61/61	1/1	72/72	5/5	67/67
		(43.9%, 100.0%)	(93.0%, 99.8%)	(90.8%, 100.0%)	(88.9%, 99.6%)	(74.1%, 100.0%)	(94.1%, 100.0%)	(20.7%, 100.0%)	(94.9%, 100.0%)	(56.6%, 100.0%)	(94.6%, 100.0%)
	Total	93.0%	99.2%	100.0%	99.2%	97.1%	100.0%	86.5%	99.1%	92.5%	99.8%
		80/86	940/948	69/69	964/972	33/34	998/998	45/52	961/970	49/53	979/981
		(85.6%, 96.8%)	(98.3%, 99.6%)	(94.7%, 100.0%)	(98.4%, 99.6%)	(85.1%, 99.5%)	(99.6%, 100.0%)	(74.7%, 93.3%)	(98.2%, 99.5%)	(82.1%, 97.0%)	(99.3%, 99.9%)
Unpreserved	In-patient	93.8%	99.5%	100.0%	99.8%	100.0%	99.8%	64.3%	99.8%	60.0%	99.8%
		30/32	414/416	10/10	435/436	4/4	439/440	9/14	422/423	3/5	433/434
		(79.9%, 98.3%)	(98.3%, 99.9%)	(72.2%, 100.0%)	(98.7%, 100.0%)	(51.0%, 100.0%)	(98.7%, 100.0%)	(38.8%, 83.7%)	(98.7%, 100.0%)	(23.1%, 88.2%)	(98.7%, 100.0%)
	Out-patient	80.0%	99.4%	100.0%	100.0%	75.0%	100.0%	100.0%	100.0%	100.0%	99.4%
		4/5	178/179	11/11	188/188	3/4	184/184	11/11	177/177	14/14	174/175
		(37.6%, 96.4%)	(96.9%, 99.9%)	(74.1%, 100.0%)	(98.0%, 100.0%)	(30.1%, 95.4%)	(98.0%, 100.0%)	(74.1%, 100.0%)	(97.9%, 100.0%)	(78.5%, 100.0%)	(96.8%, 99.9%)

		Norovirus		Rotavirus		Adenovirus		Sapovirus		Astrovirus	
		**PPA**	**NPA**	**PPA**	**NPA**	**PPA**	**NPA**	**PPA**	**NPA**	**PPA**	**NPA**
**Specimen Type**	**Patient Population**	**(95% CI)**	**(95% CI)**	**(95% CI)**	**(95% CI)**	**(95% CI)**	**(95% CI)**	**(95% CI)**	**(95% CI)**	**(95% CI)**	**(95% CI)**
	Emergency	94.4%	100.0%	100.0%	100.0%	100.0%	100.0%	88.9%	99.2%	100.0%	99.2%
		17/18	110/110	9/9	123/123	1/1	131/131	8/9	122/123	13/13	119/120
		(74.2%, 99.0%)	(96.6%, 100.0%)	(70.1%, 100.0%)	(97.0%, 100.0%)	(20.7%, 100.0%)	(97.2%, 100.0%)	(56.5%, 98.0%)	(95.5%, 99.9%)	(77.2%, 100.0%)	(95.4%, 99.9%)
	Long Term Care Facility	No data for PPA calculation	100.0%	100.0%	100.0%	No data for PPA calculation	100.0%	No data for PPA calculation	100.0%	No data for PPA calculation	100.0%
			5/5	1/1	5/5		5/5		5/5		5/5
			(56.6%, 100.0%)	(20.7%, 100.0%)	(56.6%, 100.0%)		(56.6%, 100.0%)		(56.6%, 100.0%)		(56.6%, 100.0%)
	Unknown	92.0%	100.0%	100.0%	96.9%	100.0%	100.0%	No data for PPA calculation	100.0%	100.0%	100.0%
		23/25	45/45	36/36	31/32	2/2	56/56		33/33	1/1	36/36
		(75.0%, 97.8%)	(92.1%, 100.0%)	(90.4%, 100.0%)	(84.3%, 99.4%)	(34.2%, 100.0%)	(93.6%, 100.0%)		(89.6%, 100.0%)	(20.7%, 100.0%)	(90.4%, 100.0%)
	Total	92.5%	99.6%	100.0%	99.7%	90.9%	99.9%	82.4%	99.7%	93.9%	99.6%
		74/80	752/755	67/67	782/784	10/11	815/816	28/34	759/761	31/33	767/770
		(84.6%, 96.5%)	(98.8%, 99.9%)	(94.6%, 100.0%)	(99.1%, 99.9%)	(62.3%, 98.4%)	(99.3%, 100.0%)	(66.5%, 91.7%)	(99.0%, 99.9%)	(80.4%, 98.3%)	(98.9%, 99.9%)
Combined	In-patient	94.6%	99.4%	100.0%	99.9%	85.7%	99.9%	73.9%	99.6%	85.7%	99.7%
		53/56	648/652	19/19	689/690	6/7	698/699	17/23	674/677	12/14	686/688
		(85.4%, 98.2%)	(98.4%, 99.8%)	(83.2%, 100.0%)	(99.2%, 100.0%)	(48.7%, 97.4%)	(99.2%, 100.0%)	(53.5%, 87.5%)	(98.7%, 99.8%)	(60.1%, 96.0%)	(98.9%, 99.9%)
	Out-patient	90.2%	99.3%	100.0%	99.1%	95.7%	100.0%	88.5%	99.2%	94.0%	99.7%
		46/51	743/748	29/29	777/784	22/23	784/784	46/52	738/744	47/50	757/759
		(79.0%, 95.7%)	(98.4%, 99.7%)	(88.3%, 100.0%)	(98.2%, 99.6%)	(79.0%, 99.2%)	(99.5%, 100.0%)	(77.0%, 94.6%)	(98.3%, 99.6%)	(83.8%, 97.9%)	(99.0%, 99.9%)
	Emergency	93.5%	99.4%	100.0%	100.0%	100.0%	100.0%	90.0%	99.0%	93.8%	99.5%
		29/31	175/176	13/13	198/198	2/2	209/209	9/10	198/200	15/16	195/196
		(79.3%, 98.2%)	(96.9%, 99.9%)	(77.2%, 100.0%)	(98.1%, 100.0%)	(34.2%, 100.0%)	(98.2%, 100.0%)	(59.6%, 98.2%)	(96.4%, 99.7%)	(71.7%, 98.9%)	(97.2%, 99.9%)

		Norovirus		Rotavirus		Adenovirus		Sapovirus		Astrovirus	
		PPA	NPA	PPA	NPA	PPA	NPA	PPA	NPA	PPA	NPA
**Specimen Type**	**Patient Population**	**(95% CI)**	**(95% CI)**	**(95% CI)**	**(95% CI)**	**(95% CI)**	**(95% CI)**	**(95% CI)**	**(95% CI)**	**(95% CI)**	**(95% CI)**
	Long Term Care Facility	No data for PPA calculation	100.0%	100.0%	100.0%	No data for PPA calculation	100.0%	No data for PPA calculation	100.0%	No data for PPA calculation	100.0%
			5/5	1/1	5/5		5/5		5/5		5/5
			(56.6%, 100.0%)	(20.7%, 100.0%)	(56.6%, 100.0%)		(56.6%, 100.0%)		(56.6%, 100.0%)		(56.6%, 100.0%)
	Unknown	92.9%	99.2%	100.0%	97.5%	100.0%	100.0%	100.0%	100.0%	100.0%	100.0%
		26/28	121/122	74/74	77/79	13/13	117/117	1/1	105/105	6/6	103/103
		(77.4%, 98.0%)	(95.5%, 99.9%)	(95.1%, 100.0%)	(91.2%, 99.3%)	(77.2%, 100.0%)	(96.8%, 100.0%)	(20.7%, 100.0%)	(96.5%, 100.0%)	(61.0%, 100.0%)	(96.4%, 100.0%)
	Total	92.8%	99.4%	100.0%	99.4%	95.6%	99.9%	84.9%	99.4%	93.0%	99.7%
		154/166	1692/1703	136/136	1746/1756	43/45	1813/1814	73/86	1720/1731	80/86	1746/1751
		(87.8%, 95.8%)	(98.8%, 99.6%)	(97.3%, 100.0%)	(99.0%, 99.7%)	(85.2%, 98.8%)	(99.7%, 100.0%)	(75.8%, 90.9%)	(98.9%, 99.6%)	(85.6%, 96.8%)	(99.3%, 99.9%)

Table 6: Poolability

**Target**	**Performance Measurement**	**Factor**	**P-value**
Norovirus	PPA	Specimen type	0.8965
		Specimen origin	0.4542
		Test site	N/A
	NPA	Specimen type	0.2643
		Specimen origin	0.9515
		Test site	0.3196
Rotavirus	PPA	Specimen type	1.0000
		Specimen origin	1.0000
		Test site	1.0000
	NPA	Specimen type	0.1373
		Specimen origin	0.1337
		Test site	0.0049
Adenovirus	PPA	Specimen type	0.4134
		Specimen origin	0.2121
		Test site	N/A
	NPA	Specimen type	0.4498
		Specimen origin	1.0000
		Test site	0.5221
Sapovirus	PPA	Specimen type	0.5972
		Specimen origin	0.9490
		Test site	0.1053
	NPA	Specimen type	0.1054
		Specimen origin	0.8908
		Test site	0.0253
Astrovirus	PPA	Specimen type	0.7928
		Specimen origin	0.8118
		Test site	N/A
	NPA	Specimen type	0.4774
		Specimen origin	0.0187
		Test site	0.8742

N/A : Insufficient data available for statistics

Table 7: Non-Reportable Rate for Combined Target by Specimen Type, Site and Overall (MAX EVP)

**Combined Target**	**Unresolved Rate**		**Indeterminate Rate**		**Incomplete Rate**		**Total Rate**	
**Specimen Type**	**Initial EVP (95% CI)**	**Final EVP (95% CI)**	**Initial EVP (95% CI)**	**Final EVP (95% CI)**	**Initial EVP (95% CI)**	**Final EVP (95% CI)**	**Initial EVP (95% CI)**	**Final EVP (95% CI)**
Cary-Blair Preserved	0.8% 9/1085 (0.4%, 1.6%)	0.1% 1/1076 (0.0%, 0.5%)	0.5% 5/1085 (0.2%, 1.1%)	0.1% 1/1076 (0.0%, 0.5%)	0.1% 1/1085 (0.0%, 0.5%)	0.1% 1/1076 (0.0%, 0.5%)	1.4% 15/1085 (0.8%, 2.3%)	0.3% 3/1076 (0.1%, 0.8%)
Unpreserved	1.8% 18/997 (1.1%, 2.8%)	1.0% 10/982 (0.6%, 1.9%)	2.2% 22/997 (1.5%, 3.3%)	0.0% 0/982 (0.0%, 0.4%)	0.3% 3/997 (0.1%, 0.9%)	0.0% 0/982 (0.0%, 0.4%)	4.3% 43/997 (3.2%, 5.8%)	1.0% 10/982 (0.6%, 1.9%)
Combined	1.3% 27/2082 (0.9%, 1.9%)	0.5% 11/2058 (0.3%, 1.0%)	1.3% 27/2082 (0.9%, 1.9%)	0.0% 1/2058 (0.0%, 0.3%)	0.2% 4/2082 (0.1%, 0.5%)	0.0% 1/2058 (0.0%, 0.3%)	2.8% 58/2082 (2.2%, 3.6%)	0.6% 13/2058 (0.4%, 1.1%)

Table 8: Discrepant Norovirus Results

Specimen Origin	Study Number	Site	Specimen Type collected	Historical Result for Norovirus	Alt-PCR Result for Norovirus (non_seq)	Alt-PCR Result for Norovirus (seq)	Sequencing Result for Norovirus	Composite Final RM Result for Norovirus	Final Norovirus Result Status (Composite RM)	Final MAX Norovirus Result	Final Norovirus CT.score (MM1 MAX Channel 0)	Final Norovirus Cycle EP (MM1 MAX	Discrepant FilmArray GastroIntestinal Panel Result
Prospective	XC1104P	CIN	Cary-Blair Preserved	na	POS	POS	NEG	POS	FN	NEG	38.3	138	NEG
Prospective	XC1142P	CIN	Cary-Blair Preserved	na	NEG	POS	POS	POS	FN	NEG	31.4	172	NEG
Prospective	XC1149P	CIN	Cary-Blair Preserved	na	POS	POS	POS	POS	FN	NEG	36.3	190	NEG
Prospective	XC1238P	CIN	Cary-Blair Preserved	na	NEG	POS	POS	POS	FN	NEG	32.9	217	NEG
Prospective	XD0701P	POR	Cary-Blair Preserved	na	POS	NEG	na	POS	FN	NEG	0	29	NEG
Prospective	XD0946P	POR	Cary-Blair Preserved	na	POS	POS	POS	POS	FN	NEG	34.9	147	NEG
Prospective	XB0306P	CAL	Unpreserved	na	POS	NEG	na	POS	FN	NEG	0	25	na
Prospective	XB0331P	CAL	Unpreserved	na	POS	POS	NEG	POS	FN	NEG	0	49	na
Prospective	XB0347P	CAL	Unpreserved	na	NEG	POS	POS	POS	FN	NEG	39.9	118	na
Prospective	XB0368P	CAL	Unpreserved	na	NEG	POS	POS	POS	FN	NEG	0	-13	na
Retrospective	XC0152R	CIN	Unpreserved	POS	na	POS	POS	POS	FN	NEG	0	58	na
Retrospective	XG0022R	QBC	Unpreserved	POS	na	POS	POS	POS	FN	NEG	33.9	227	na
Prospective	XA0637P	ALB	Cary-Blair Preserved	na	NEG	POS	NEG	NEG	FP	POS	30.8	636	POS Noro & Astro
Prospective	XC1041P	CIN	Cary-Blair Preserved	na	NEG	POS	NEG	NEG	FP	POS	30.9	1940	POS Noro
Prospective	XC1308P	CIN	Cary-Blair Preserved	na	NEG	POS	NEG	NEG	FP	POS	31.6	2332	POS Noro
Prospective	XC1419P	CIN	Cary-Blair Preserved	na	NEG	POS	NEG	NEG	FP	POS	33.1	280	POS Adeno
Prospective	XD0883P	POR	Cary-Blair Preserved	na	NEG	POS	NEG	NEG	FP	POS	32.9	560	POS Noro
Prospective	XF0510P	JHU	Cary-Blair Preserved	na	NEG	POS	NEG	NEG	FP	POS	28.6	3881	NEG
Prospective	XF0544P	JHU	Cary-Blair Preserved	na	NEG	POS	NEG	NEG	FP	POS	28.6	4698	NEG
Retrospective	XG0049R	QBC	Cary-Blair Preserved	NEG	na	POS	NEG	NEG	FP	POS	28.4	4891	na
Prospective	XB0564P	CAL	Unpreserved	na	NEG	POS	NEG	NEG	FP	POS	35.7	520	na
Prospective	XB0916P	CAL	Unpreserved	na	NEG	POS	NEG	NEG	FP	POS	32.6	1955	na
Prospective	XF0466P	JHU	Unpreserved	na	NEG	POS	NEG	NEG	FP	POS	31	1288	na

Table 9: Discrepant Rotavirus Results

Specimen Origin	Study Number	Site	Specimen Type collected	Historical Result for Rotavirus	Alt-PCR Result for Rotavirus (non_seq)	Alt-PCR Result for Rotavirus (seq)	Sequencing Result for Rotavirus	Composite Final RM Result for Rotavirus	Final Rotavirus Result Status (Composite RM)	Final MAX Rotavirus Result	Rotavirus CT.score (MM1 MAX Channel 3)	Final Rotavirus Cycle EP (MM1 MAX Channel 3)	Discrepant FilmArray GastroIntestinal Panel Result
Prospective	XC1273P	CIN	Cary-Blair Preserved	na	NEG	NEG	na	NEG	FP	POS	33.6	244	POS Sapo
Prospective	XC1274P	CIN	Cary-Blair Preserved	na	NEG	NEG	na	NEG	FP	POS	33.8	364	NEG
Prospective	XC1275P	CIN	Cary-Blair Preserved	na	NEG	NEG	na	NEG	FP	POS	32.1	777	POS Rota
Prospective	XC1328P	CIN	Cary-Blair Preserved	na	NEG	NEG	na	NEG	FP	POS	27.6	952	POS Rota
Prospective	XC1332P	CIN	Cary-Blair Preserved	na	NEG	NEG	na	NEG	FP	POS	28.1	677	POS Rota & Noro
Prospective	XC1337P	CIN	Cary-Blair Preserved	na	NEG	POS	NEG	NEG	FP	POS	26.8	1107	POS Rota, Noro & Astro
Prospective	XC1370P	CIN	Cary-Blair Preserved	na	NEG	NEG	na	NEG	FP	POS	29.6	718	POS Sapo & Astro
Retrospective	XG0027R	QBC	Cary-Blair Preserved	NEG	na	NEG	na	NEG	FP	POS	33.6	352	na
Prospective	XE0377P	LUR	Unpreserved	na	NEG	POS	NEG	NEG	FP	POS	30.8	829	na
Retrospective	XG0041R	QBC	Unpreserved	NEG	na	POS	NEG	NEG	FP	POS	28.2	1400	na

Table 10: Discrepant Astrovirus Results

Specimen Origin	Study Number	Site	Specimen Type collected	Historical Result for Astrovirus	Alt-PCR Result for Astrovirus (non_seq)	Alt-PCR Result for Astrovirus (seq)	Sequencing Result for Astrovirus	Composite Final RM Result for Astrovirus	Astrovirus Result Status (Composite RM)	Final MAX Astrovirus Result	Astrovirus CT.score (MM2 MAX Channel 0)	Astrovirus Cycle EP (MM2 MAX Channel 0)	Discrepant FilmArray GastroIntestinal Panel Result
Prospective	XC1132P	CIN	Cary-Blair Preserved	na	POS	NEG	na	POS	FN	NEG	0	-19	NEG
Prospective	XC1138P	CIN	Cary-Blair Preserved	na	POS	NEG	na	POS	FN	NEG	36.4	73	POS Adeno
Retrospective	XC0894P	CIN	Cary-Blair Preserved	POS	na	POS	POS	POS	FN	NEG	0	25	na
Retrospective	XC0913P	CIN	Cary-Blair Preserved	POS	na	POS	POS	POS	FN	NEG	35.6	99	na
Prospective	XB0824P	CAL	Unpreserved	na	POS	NEG	na	POS	FN	NEG	0	-24	na
Prospective	XE0345P	LUR	Unpreserved	na	POS	NEG	na	POS	FN	NEG	0	-8	na
Prospective	XD0718P	POR	Cary-Blair Preserved	na	NEG	NEG	na	NEG	FP	POS	26.5	195	NEG
Retrospective	XC0678P	CIN	Cary-Blair Preserved	NEG	na	NEG	na	NEG	FP	POS	35.3	423	na
Prospective	XB0636P	CAL	Unpreserved	na	NEG	NEG	na	NEG	FP	POS	33.1	239	na
Prospective	XF0342P	JHU	Unpreserved	na	NEG	NEG	na	NEG	FP	POS	27.6	198	na
Retrospective	XB0155P	CAL	Unpreserved	NEG	na	NEG	na	NEG	FP	POS	25.9	399	na

Table 11: Discrepant Sapovirus Results

Specimen Origin	Study Number	Site	Specimen Type collected	Historical Result for Sapovirus	Alt-PCR Result for Sapovirus (non_seq)	Alt-PCR Result for Sapovirus (seq)	Sequencing Result for Sapovirus	Composite Final RM Result for	Final Sapovirus Result Status	Final MAX Sapovirus Result	Final Sapovirus CT.score	Final Sapovirus Cycle EP	Discrepant FilmArray GastroIntestinal
Prospective	XC1109P	CIN	Cary-Blair Preserved	na	POS	POS	NEG	POS	FN	NEG	0	19	POS Rota & Astro
Prospective	XC1319P	CIN	Cary-Blair Preserved	na	POS	POS	POS	POS	FN	NEG	0	32	POS Sapo
Prospective	XD0634P	POR	Cary-Blair Preserved	na	POS	POS	na	POS	FN	NEG	0	18	NEG
Prospective	XD0865P	POR	Cary-Blair Preserved	na	POS	NEG	na	POS	FN	NEG	0	12	NEG
Prospective	XD0910P	POR	Cary-Blair Preserved	na	POS	POS	POS	POS	FN	NEG	0	15	POS Sapo & Adeno
Prospective	XD0951P	POR	Cary-Blair Preserved	na	POS	NEG	na	POS	FN	NEG	0	13	NEG
Retrospective	XC0004P	CIN	Cary-Blair Preserved	POS	na	POS	POS	POS	FN	NEG	0	-62	na
Prospective	XB0398P	CAL	Unpreserved	na	POS	POS	na	POS	FN	NEG	32.3	65	na
Prospective	XB0402P	CAL	Unpreserved	na	POS	NEG	na	POS	FN	NEG	0	5	na
Prospective	XB0403P	CAL	Unpreserved	na	POS	POS	NEG	POS	FN	NEG	0	4	na
Prospective	XB0409P	CAL	Unpreserved	na	POS	POS	NEG	POS	FN	NEG	0	11	na
Prospective	XB0770P	CAL	Unpreserved	na	POS	POS	POS	POS	FN	NEG	35.6	68	na
Prospective	XE0345P	LUR	Unpreserved	na	POS	POS	POS	POS	FN	NEG	0	12	na
Prospective	XA0685P	ALB	Cary-Blair Preserved	na	NEG	POS	NEG	NEG	FP	POS	31	159	POS Sapo
Prospective	XC1059P	CIN	Cary-Blair Preserved	na	NEG	NEG	na	NEG	FP	POS	32.8	219	POS Sapo
Prospective	XC1060P	CIN	Cary-Blair Preserved	na	NEG	POS	NEG	NEG	FP	POS	28.9	723	POS Sapo
Prospective	XC1066P	CIN	Cary-Blair Preserved	na	NEG	NEG	na	NEG	FP	POS	33.5	450	POS Sapo & Noro
Prospective	XC1080P	CIN	Cary-Blair Preserved	na	NEG	NEG	na	NEG	FP	POS	33.1	139	NEG
Prospective	XC1266P	CIN	Cary-Blair Preserved	na	NEG	POS	NEG	NEG	FP	POS	31.8	341	NEG
Prospective	XC1360P	CIN	Cary-Blair Preserved	na	NEG	NEG	na	NEG	FP	POS	30.5	546	POS Noro
Prospective	XC1391P	CIN	Cary-Blair Preserved	na	NEG	NEG	na	NEG	FP	POS	33.8	148	NEG
Prospective	XF0377P	JHU	Cary-Blair Preserved	na	NEG	POS	NEG	NEG	FP	POS	36.2	175	NEG
Prospective	XE0398P	LUR	Unpreserved	na	NEG	NEG	na	NEG	FP	POS	34.8	192	na
Retrospective	XE0205P	LUR	Unpreserved	NEG	na	POS	NEG	NEG	FP	POS	29.5	1203	na

Table 12: Discrepant Adenovirus Results

Specimen Origin	Study Number	Site	Specimen Type collected	Historical Result for Adenovirus	Alt-PCR Result for Adenovirus (non_seq)	Alt-PCR Result for Adenovirus (seq)	Sequencing Result for Adenovirus	Composite Final RM Result for Adenovirus	Final Adenovirus Result Status (Composite RM)	Final MAX Adenovirus Result	Final Adenovirus CT.score (MM1 MAX Channel 5)	Final Adenovirus Cycle EP (MM1 MAX Channel 5)	Discrepant FilmArray GastroIntestinal Panel Result
Prospective	XC1162P	CIN	Cary-Blair Preserved	na	NEG	POS	POS	POS	FN	NEG	0	0	na
Prospective	XB0566P	CAL	Unpreserved	na	POS	NEG	na	POS	FN	NEG	0	35	na
Prospective	XB0588P	CAL	Unpreserved	na	NEG	POS	NEG	NEG	FP	POS	37.2	113	na

Appendix A: Further details on the reference method

Reference method testing consisted of:

- Nucleic acid extraction of pre-screened, and quantified, clinical positive stool specimens, virus, viral culture fluid, or dilutions of target DNA
- Two (2) alternate rtPCR assays
- Sanger Sequencing.

Quantified samples were extracted on the Roche MagNA Pure LC 2.0 using the Total Nucleic Acid Isolation Kit – High Performance according to the instrument user manual and kit protocol. Following extraction or gBlock dilution, the purified viral nucleic acid, or synthetic DNA, was tested with different Master Mixes representing two (2) categories of assays, the non-sequencing assay group, and the sequencing assay group.

The non-sequencing assays consisted of eleven (11) TaqMan®-based real-time rtPCR assays. Detection for these assays is achieved through the use of fluorescently labeled probes. The sequencing assays group consisted of ten (10) melt-based real-time rtPCR assays. rtPCR detection was achieved through the use of Melt Temperature Analysis with an intercalating dye.

rtPCR for both sequencing and non-sequencing assays was performed on the Bio-Rad CFX96 or Bio-Rad CFX96 Touch™ Real-Time PCR Detection Systems according to the instrument Instruction Manual. For the Sequencing assay group, potentially positive samples were identified and then further processed for Sanger Sequencing. Sanger Sequencing was performed on the Advanced Biosystems® GA3500xL Genetic Analyzer according to the User Guide.

Reference Method Validation

The reference method consists of twenty-one (21) assays that test for multiple genotypes, each, of the five (5) viral targets of interest (Norovirus, Rotavirus, Adenovirus, Sapovirus, and Astrovirus). Ten (10) assays, for which, viral targets/genotypes had a previously characterized limit of detection (LoD), were utilized for this study. A representative strain of each organism target was used in this study (**Table A1**). Each strain was tested and analyzed individually. The remaining viral genotypes and assays were used to evaluate inclusivity.

LoD titration and determination was tested using a limiting dilution approach and followed by confirmation. LoD evaluation studies were performed using MagNA Pure LC 2.0 DNA extraction from a target-negative, unpreserved, stool matrix using the parameters described below. Unpreserved stool matrix represents the "worst-case" sample matrix as the enteric viral targets under investigation are more susceptible to degradation—as compared to Cary-Blair preserved samples. Therefore, both unpreserved and Cary-Blair preserved specimen types were considered validated through this study.

Amplification and detection were performed using a total of four (4) CFX96™ platforms. Ct Score, EP (fluorescence height—end of the reaction curve) and baseline values were generated using the Bio-Rad CFX Manager (version 2 or later) software.

Acceptance Criteria

Twenty (20) replicates were tested to confirm the putative LoD for each strain. LoD confirmation was achieved when the proportion positive was ≥ 95%. No further testing was performed if LoD confirmation resulted in 95% (19/20) proportion positive during initial confirmation testing. If the proportion positive was below 95%, additional testing at a higher level was required.

External positive and negative controls were included on each extraction run, subsequent PCR amplification and sequencing (when applicable), and were required to meet expected results for a positive sample to be considered valid. Successful sample extraction was required on the MagNA Pure LC 2.0.

For a potentially positive sequenced sample to be considered positive for its intended assay target, it was required to achieve the following criteria:

- Sample score QV20 ≥ 90% for both the forward and reverse sequence reads; and
- BLAST E-value of the consensus read ≤ 10-30 with at least 90% query coverage and 95% identify for the intended assay target.

Conclusion

Limit of detection (LoD) analysis was completed for 21 master mixes used in the sequencing and non-sequencing reference method assays (inclusive for all five viral targets). **Table A2** lists the final reference method validation results in cp/mL. All reference method assays passed initial testing with a proportion positive of ≥ 95%, except for Master Mix C (Norovirus), which required testing at a higher concentration. All acceptance criteria were met for the reference method validation, demonstrating that the EVP alternate PCR reference method assays and workflow were able to correctly detect and identify Norovirus, Rotavirus, Adenovirus, Sapovirus and Astrovirus in the presence of unpreserved stool specimens. The validated reference method was deemed acceptable for use in the clinical trial.

Analytical Reactivity (Inclusivity)

The objective was to demonstrate the ability of the sequencing and non-sequencing reference method assays to detect various strains of Norovirus, Rotavirus, Adenovirus, Sapovirus and Astrovirus representing geographic, temporal and phylogenetic diversity.

A variety of sequencing and non-sequencing reference method assay target strains were included in these analyses. Strain selection criteria included prevalence, genotype and geographic diversity, where appropriate. The analytical reactivity of the BD MAX EVP Alternate PCR Reference Method was tested using clinical positive stool samples. For assays in which a clinical positive stool sample was unable to be identified, the assay was tested using a synthetic, custom made, double-stranded DNA gene fragment (gBlock)

Quantified samples were extracted on the Roche MagNA Pure LC 2.0 using the Total Nucleic Acid Isolation Kit – High Performance according to the instrument user manual and kit protocol. Following extraction or gBlock dilution, the purified viral nucleic acid, or synthetic DNA, was tested with different Master Mixes representing two (2) categories of assays, the non-sequencing assay group, and the sequencing assay group.

The non-sequencing assays group consisted of eleven (11) TaqMan®-based real-time rtPCR assays. Detection for these assays is achieved through the use of fluorescently labeled probes.

The sequencing assays group consisted of ten (10) melt-based real-time rtPCR assays. rtPCR detection was achieved through the use of Melt Temperature Analysis with an intercalating dye. Each individual assay had an evaluation of analytical reactivity, resulting in a distinct value for each target genotype. rtPCR for both sequencing and non-sequencing assays was performed on the Bio-Rad CFX96 or Bio-Rad CFX96 Touch™ Real-Time PCR Detection Systems according to the instrument Instruction Manual. For the Sequencing assay group, potentially positive samples were identified and then further processed for Sanger Sequencing. Sanger Sequencing was performed on the Advanced Biosystems® GA3500xL Genetic Analyzer according to the User Guide.

A total of 70 specimens comprised of viral and gBlock targets were evaluated in the study. Each strain was initially tested in triplicate. If the required proportion positive percentage (100% for three replicates) failed to be met, the target(s) of interest were titrated higher until the proportion positive was ≥95%.

Alternative PCR Inclusivity non-sequencing

Sixty-one (61) of the 70 samples were available for testing on non-sequencing assays, of which there were 41 samples of that confirmed at ≥95% at 3x LoD. The remaining samples were titrated upwards. Seven (7) more were confirmed at 30x LoD, five (5) more were confirmed at 300x LoD, and four (4) more required titration to 3000x LoD. The remaining four (4) samples were not detected. A Summary of the non-sequencing results can be found in **Table A3**.

Alternative PCR Inclusivity sequencing

Sixty (60) of the 70 samples were available for testing on sequencing assays, of which there were 39 samples that confirmed at 3x LoD. The remaining samples were then titrated up to 30x LoD where seven (7) more samples were confirmed. Nine (9) samples confirmed at 300x LoD, and finally two (2) were confirmed at 3000x LoD. The remaining three (3) samples were not detected. A Summary of the non-sequencing results can be found in **Table A4.**

The EVP Alternate PCR Reference Method assays were shown to be inclusive of all tested genotypes.

Table A1. Non-sequencing reference method assays

Non-sequencing		
**Target Virus**	**Assay ID**	**Target Genotype**
Norovirus	C[a]	GII.1, GII.3, GII.4, GII.12
	E	GI.3, GI.6, GI.7
	F	GI.4
	H	GII.6
Rotavirus	U[b]	Type A
Adenovirus	R[c]	Type F40/41
Sapovirus	K[d]	GI
	J	GII
	M	GIV, GV
Astrovirus	OQ[e]	Type 1-8, MLB 1-3
	P	HMO-VA 1-2

Sequencing		
**Target Virus**	**Assay ID**	**Target Genotype**
Norovirus	AC	GI
	Y[a]	GII.4, GII.6
	AB	GII.2, GII.2, GII.3, GII.12, GII.17
Rotavirus	T[b]	Type A
Adenovirus	S[c]	Type F40/41
Sapovirus	X	GII
	V	GIV
	W	GV
	I[d]	GI
Astrovirus	N[e]	Type 1-8, MLB 1-3

[a]GII.4 used for reference method testing during study execution; source was positive clinical stool
[b]Va70 used for reference method testing during study execution; source was ZeptoMetrix viral culture fluid
[c]Type F40 used for reference method testing during study execution; source was ZeptoMetrix viral culture fluid
[d]GI used for reference method testing during study execution; source was positive clinical stool
[e]Type 8 used for reference method testing during study execution; source was ZeptoMetrix viral culture fluid

Table A2. Summary of Reference Method Validation Results

Assay Type	Assay	Virus	Concentration (cp/mL)	Results
Non-Sequencing	C[a]	Norovirus	1.56×10^4	100% (20/20)
	C	Norovirus	1.56×10^3	90% (18/20)
Non-Sequencing	R	Adenovirus	4.49×10^4	100% (20/20)
Non-Sequencing	K	Sapovirus	3.25×10^5	100% (20/20)
Non-Sequencing	OQ	Astrovirus	6.89×10^4	100% (20/20)
Non-Sequencing	U	Rotavirus	2.09×10^4	100% (20/20)
Sequencing	Y	Norovirus	2.02×10^4	100% (20/20)
Sequencing	S	Adenovirus	4.49×10^6	100% (20/20)
Sequencing	I	Sapovirus	3.25×10^6	95% (19/20)
Sequencing	N	Astrovirus	6.89×10^6	100% (20/20)
Sequencing	T	Rotavirus	2.09×10^5	95% (19/20)

[a]Master-Mix C reaction failed at 1.56×10^3 and was repeated at 1.56×10^4

Table A3. Alternative PCR inclusivity—non-sequencing assay results

Virus	Assay ID(Non-sequencing)	Type	Source	Strains Tested
Adenovirus	R	F40/F41	Clinical Stool	6[a]
Astrovirus	OQ	1	Viral Stock	1
		2	Viral Stock	1
		3	Viral Stock	1
		4	Viral Stock	2[b]
		5	Viral Stock	1
		6	Viral Stock	1
		7	Viral Stock	1
		8	Viral Stock	1
		Unknown	Clinical Stool	1
		MLB 1	gBlock	2[c]
		MLB 2	gBlock	1[d]
		MLB 3	gBlock	1[e]
	P	VA1	gBlock	1
		VA2	gBlock	1
		HMO	gBlock	1
Norovirus GI	E	3	gBlock	2[f]
		6	Clinical Stool	1[g]
		7	Clinical Stool	1[h]
			gBlock	1
	F	4	Clinical Stool	1
Norovirus GII	C	1	Clinical Stool	2[i]
		3	Clinical Stool	2[J]
		4	Clinical Stool	8[k]
		12	Clinical Stool	2[L]
		17	Clinical Stool	1[m]
	H	6	Clinical Stool	3[n]
Rotavirus	U	Unknown	Clinical Stool	1
		Type A	Viral Stock	2
Sapovirus	J	GII	Clinical Stool	2[o]
	K	GI	Clinical Stool	6
	M	GIV	Clinical Stool	3

[a]Two Adenovirus F40/41 clinical stools were detected at 30x LOD.
[b]One Astrovirus, Type 4 (Type 4 ERE IID 1358 lot 313555), was detected at 30X LOD.
[c]One Astrovirus MLB1 gBlock was detected at 3000x LOD.
[d]One Astrovirus MLB2 gBlock was detected at 30x LOD.
[e]One Astrovirus MLB3 gBlock was detected at 3000x LOD.
[f]Both Norovirus GI.3 gBlocks were detected at 3000x LOD.
[g]One Norovirus GI.6 clinical stool was detected at 0.4x LOD.
[h]One Norovirus GI.7 (Guix NoV GI.7 10.31750) clinical stool was not detected at 7x LOD.
[i]Both Norovirus GII.1 clinical stool were detected at 300x LOD.
[J]Both Norovirus GII.3 clinical stool were detected at 300x LOD.
[k]One Norovirus GII.4 clinical stool was detected at 3x LOD.
[L]One Norovirus GII.12 clinical stool was detected at 300x LOD. The other Norovirus GII.12 clinical stool was tested at 1692x LOD and was not detected.
[m]One Norovirus GII.17 clinical stool was not detected at 300x LOD.
[n]One Norovirus GII.6 clinical stool was detected at 30x LOD.
[o]One Sapovirus GII clinical stool was detected at 30x LOD. The other Sapovirus GII clinical stool was tested at 3021x LOD and was not detected.

Table A4. Alternative PCR inclusivity—sequencing assay results

Virus	Assay ID(Non-sequencing)	Type	Source	Strains Tested
Adenovirus	S	F40/F41	Clinical Stool	6[a]
Astrovirus	N	1	Viral Stock	1
		2	Viral Stock	1
		3	Viral Stock	1
		4	Viral Stock	2
		5	Viral Stock	1
		6	Viral Stock	1
		7	Viral Stock	1[b]
		8	Viral Stock	1
		Unknown	Clinical Stool	1
		MLB 1	gBlock	1[c]
		MLB 2	gBlock	1[d]
		MLB 3	gBlock	1[e]
Norovirus GI	AC	3	gBlock	2[f]
		4	Clinical Stool	1
		6	Clinical Stool	1
		7	Clinical Stool	1[g]
Norovirus GII	AB	1	Clinical Stool	2[h]
		2	Clinical Stool	3
		3	Clinical Stool	2[i]
		12	Clinical Stool	2[J]
		17	Clinical Stool	2[k]
	Y	4	Clinical Stool	8
		6	Clinical Stool	3[L]
Rotavirus	T	Unknown	Clinical Stool	1[m]
		Type A	Viral Stock	2[n]
Sapovirus	I	GI	Clinical Stool	6[o]
	X	GII	Clinical Stool	2[p]
	V	GIV	Clinical Stool	2[q]
	W	GV	Clinical Stool	1

[a]Four Adenovirus F40/41 clinical stools were detected at higher concentrations, 104x LOD, 234x LOD, 255x LOD, and 982x LOD.
[b]One Astrovirus Type 7 viral stock was not detected at 528x LOD.
[c]One Astrovirus MLB1 gBlock was detected at 30x LOD.
[d]One Astrovirus MLB2 gBlock was detected at 30x LOD.
[e]One Astrovirus MLB3 gBlock was detected at 30x LOD.
[f]One Norovirus GI.3 gBlock was detected at 300x LOD and another GI.3 gBlock was detected at 3000x LOD.
[g]One Norovirus GI.7 clinical stool was detected at 30x LOD.
[h]One Norovirus GII.1 clinical stool was detected at 30x LOD.
[i]One Norovirus GII.3 clinical stool was detected at 300x LOD.
[J]One Norovirus GII.12 clinical stool was detected at 30x LOD.
[k]One Norovirus GII.17 clinical stool was detected at 30x LOD.
[L]One Norovirus GII.6 clinical stool was detected at 0.9x LOD.
[m]One Rotavirus (unknown) clinical stool was detected at 300x LOD.
[n]One Rotavirus A viral stock was detected at 54x LOD.
[o]One Sapovirus GI clinical stool was detected at a concentration of 2.5x LOD; one Sapovirus GI clinical stool was detected at a concentration of 57x LOD; one Sapovirus GI clinical stool was not detected at a concentration of 2529x LOD.
[p]One Sapovirus GII clinical stool was not detected at 302x LOD.
[q]One Sapovirus GIV clinical stool was detected at 300x LOD.

Appendix B: Repeat strategy

Any sample included in a run where an external control failed, or any sample for which an unresolved result, indeterminate result, or incomplete result was obtained was retested along with external controls according to the following repeat strategy:

- The first repeat was performed from the BD MAX Sample Buffer Tube (SBT).
- If required, a second repeat was performed from the stool Specimen; a new BD MAX SBT was inoculated;
- If required, a third repeat was performed from the second BD MAX SBT.

Table B1. Total Non-Reportable Rate for Combined Target by Specimen Type and Overall (BD MAX EVP)

**Combined Target**	**Non-Reportable (INC/IND/UNR) Rate**			
**Specimen Type**	**Initial**	**After 1 Repeat If Available**	**After 2 Repeats If Available**	**After 3 Repeats If Available**
Cary-Blair Preserved	1.4% (15/1085)	0.7% (8/1085)	0.5% (5/1085)	0.5% (5/1085)
Unpreserved	4.3% (43/997)	1.7% (17/997)	1.5% (15/997)	1.5% (15/997)
Combined	2.8% (58/2082)	1.2% (25/2082)	1.0% (20/2082)	1.0% (20/2082)

INC: incomplete, IND: Indeterminate, UNR: unresolved

Appendix C: Non-evaluable specimen definitions and numbers

There are two categories of RM non-evaluable: (1) due to no historical result or missing information for one more target virus or (2) due to inability to confirm the archival result with the reference method.

Table C1. Breakdown of "RM non-evaluable" specimens that were excluded from analysis.

	**Non-evaluable category**	**Norovirus**	**Astrovirus**	**Sapovirus**	**Rotavirus**	**Adenovirus**
A	No Historical results	119	294	195	85	133
B	No archival confirmation with specimen history	59	18	37	70	55
C	Total	178	212	232	155	188
	RM non-evaluable rate [(A+B)/C] involving "no history"	.087	.103	.113	.076	.092
	RM non-evaluable rate (B/C) not involving "no history"	.029	.009	.018	.034	.027
